# Microfluidic Chip Platforms for Red Blood Cell Storage Lesion Quality Control and Precision Transfusion

**DOI:** 10.34133/research.1405

**Published:** 2026-08-18

**Authors:** Yiting Lei, Yunbo Tian, Junhong Yang, Bujin Liu, Haiman Zou, Charlotte A. E. Hauser, Xia Huang, Zhong Alan Li, Danli Cui

**Affiliations:** ^1^ Department of Orthopaedic Surgery, Chongqing Municipal Health Commission Key Laboratory of Musculoskeletal Regeneration and Translational Medicine, Chongqing Municipal Engineering Research Center of Higher Education Institutions of Orthopaedic Innovation and Translation, The First Affiliated Hospital of Chongqing Medical University, Chongqing 400016, China.; ^2^ Department of Biomedical Engineering, The Chinese University of Hong Kong, Hong Kong SAR 999077, China.; ^3^ Chongqing Blood Center, Chongqing 400015, China.; ^4^ Institute of Health Care Engineering with European Testing Center of Medical Devices, Graz University of Technology, Graz 8010, Austria.

## Abstract

Red blood cell (RBC) transfusion is a core clinical intervention. However, hypothermic storage induces progressive biochemical, structural, and functional impairments collectively termed the RBC storage lesion (RSL), which compromises post-transfusion efficacy and safety. Conventional RSL detection relies on bulk population-averaged indicators with low physiological relevance and no single-cell resolution, failing to capture cellular heterogeneity and microcirculatory dysfunction. Lab-on-a-chip (LOC) platforms are established with microfluidic technology, and organ-on-a-chip represents a biomimetic and advanced extension of such systems. Together, they enable biomimetic reconstruction of the in vivo microcirculatory microenvironment, high-throughput single-cell analysis, and quantitative assessment of RBC mechanical phenotypes under physiological shear conditions. Distinct from fragmented prior reviews that separate microfluidic engineering from transfusion clinical demands, this work systematically outlines the molecular mechanisms and clinical impacts of RSL alongside unresolved detection bottlenecks, and builds an integrated LOC–endothelium-on-a-chip technical framework covering structural design, biocompatible material screening, and standardized fabrication workflows. It further summarizes microfluidic core applications including single-cell deformability quantification, stiffness evaluation, hemolysis susceptibility testing, microvascular occlusion simulation, and RBC–endothelial interaction analysis. Uniquely, it constructs a tiered translational roadmap integrating microfluidics with multi-omics, artificial intelligence, vascularized multi-organ chips, and function-centered pre-transfusion surveillance, and proposes microfluidic strategies to optimize RBC storage regimens. In summary, microfluidic technology overcomes the limitations of conventional assays for RSL quality control, provides an emerging technical platform for RSL mechanistic research, pretransfusion quality evaluation, and donor-specific precise matching, and promotes the transformation of transfusion medicine from time-based empirical management to function-oriented precision practice.

## Introduction

Red blood cell (RBC; also known as erythrocyte) transfusion is one of the most frequently performed medical interventions in clinical practice, playing a fundamental role in improving tissue oxygen supply and saving lives in trauma resuscitation, surgery, intensive care, and the treatment of various hematological disorders [1]. To meet clinical demands for large-volume blood product supply across time and space, clinical blood supply systems rely on hypothermic storage (1 to 6 °C) of RBCs in additive solutions for up to 35 to 42 d [2]. While this storage strategy resolves supply–demand contradictions and ensures transfusion accessibility, the ex vivo hypothermic storage of RBCs—placed outside the donor’s circulation—inevitably induces cumulative biological alterations collectively referred to as the RBC storage lesion (RSL) [3].

During storage, RBCs develop substantial time-dependent biochemical and structural abnormalities, including progressive depletion of adenosine triphosphate (ATP) and 2,3-diphosphoglycerate (2,3-DPG), oxidative damage to membrane lipids and proteins, decreased deformability, phospholipid externalization, and microvesicle release, accompanied by accumulation of free hemoglobin and iron. These alterations impair the ability of stored RBCs to traverse the microcirculation and modify their interactions with the endothelium and immune system [4]. As storage duration extends, free hemoglobin released from RBCs efficiently scavenges nitric oxide (NO) and, through oxidative stress mediated by free heme and non-transferrin-bound iron, activates endothelial and innate immune pathways, ultimately leading to vasoconstriction, enhanced neutrophil adhesion, microthrombosis, and impaired organ perfusion [4,5].

**Fig. 1.**
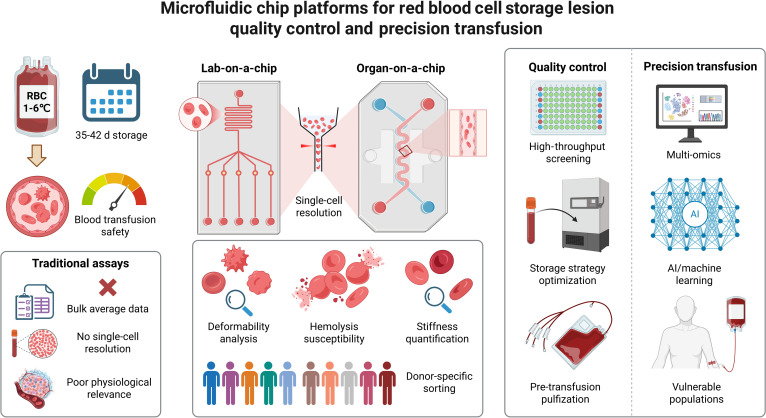
Microfluidic chips for RSL quality control and precision transfusion. Schematic illustrating the framework of microfluidic chip (LOC and OOC) applications in quality control of RSL and precision transfusion. It highlights the limitations of traditional assays, core advantages of microfluidic platforms, and key outcomes under standard hypothermic storage conditions. Created in BioRender. Lei, Y. (2026) https://BioRender.com/ryvwgzw.

Currently, fundamental research and clinical quality control regarding RSL remain predominantly dependent on bulk rheological measurements, ektacytometry, and morphological and biochemical indices. These techniques typically yield population-averaged readings under simplified shear fields or static conditions, rendering them insufficient for comprehensively characterizing the functional status of stored RBCs and predicting their post-transfusion performance [6,7]. To address these limitations, high-throughput platforms capable of characterizing mechanical and rheological phenotypes of stored RBCs at single-cell resolution under near-physiological shear conditions and complex geometries have emerged as critical technological approaches [8]. As a core platform for biomimetic microscale fluid analysis, microfluidic chips can simulate the physiological shear environment and complex geometries of in vivo microcirculation, enabling high-throughput, single-cell precision analysis of the mechanics and rheological phenotypes of stored RBCs. This provides unprecedented technical support for elucidating single-cell mechanisms underlying RSL, accurately assessing pretransfusion functional status of RBCs, optimizing blood bank storage strategies, and enhancing clinical transfusion safety [7,9,10] (Fig. 1).

Nevertheless, existing review papers lack systematic, full-coverage collation of microfluidic chip design, material matching, and manufacturing processes tailored to RSL testing. In addition, prior summaries fail to comprehensively link microfluidic single-cell biophysical readouts to the multi-layer molecular mechanisms and clinical adverse outcomes of RSL, and rarely integrate cross-cutting technologies such as multi-omics and artificial intelligence (AI) alongside forward-looking translational schemes including multi-organ biomimetic systems and functional pre-transfusion quality management. Grounded in the research progress documented in current literature, this review fills the above gaps and delivers targeted innovations: It systematically sorts microfluidic testing functions covering RBC mechanical phenotyping, hemolysis detection, and microcirculation occlusion simulation; it further explores the translational value of microfluidics in donor-specific blood screening and refined storage strategy improvement.

## Overview of RSL

RSL refers to the combined, time-dependent pathological changes in RBCs occurring at multiple levels—including structure, membrane skeleton, metabolism, and function—during routine hypothermic storage in blood banks with additive solutions [2]. Morphologically, preserved RBCs undergo a progressive transformation from the characteristic biconcave discoid morphology toward echinocytes, spheroechinocytes, and ultimately nearly spherocytic forms, accompanied by progressive reduction in surface area and declining surface area-to-volume ratios [11]. This morphological evolution exhibits strong chronological dependence, with extended storage correlated with increasing RBC density, rising osmotic fragility, and progressively declining deformability—parameters quantifiable via rheological methodologies and collectively indicating progressive deterioration of RBC mechanical properties [11]. Furthermore, storage triggers continuous microvesiculation, characterized by exponential changes in both the quantity and biochemical composition of released vesicles as storage duration progresses. Notably, microvesicles in the advanced storage phase exhibit characteristics highly similar to those generated by intracellular Ca^2+^ loading in vivo, suggesting a temporal convergence between the progression of storage-induced damage and physiological RBC aging [12].

### Key mechanisms of RSL

#### Membrane and cytoskeletal remodeling

During storage, the RBC membrane skeleton undergoes profound structural reorganization, encompassing conformational alterations in spectrin, ankyrin, and band 3 protein complexes, resulting in compromised membrane stability and increased mechanical fragility [11]. In RBCs subjected to prolonged storage under CPDA-1 (citrate phosphate dextrose adenine) conditions, progressive accumulation of cytoskeleton-bound hemoglobin monomers, multimers, and high-molecular weight crosslinked aggregates becomes detectable. Concurrently, the oxidative index of cytoskeletal proteins, particularly spectrin carbonylation, demonstrates a marked elevation from approximately day 10 of storage, with further exacerbation observed after day 35, indicative of progressive oxidative injury to the membrane skeleton induced by storage [13].

**Fig. 2.**
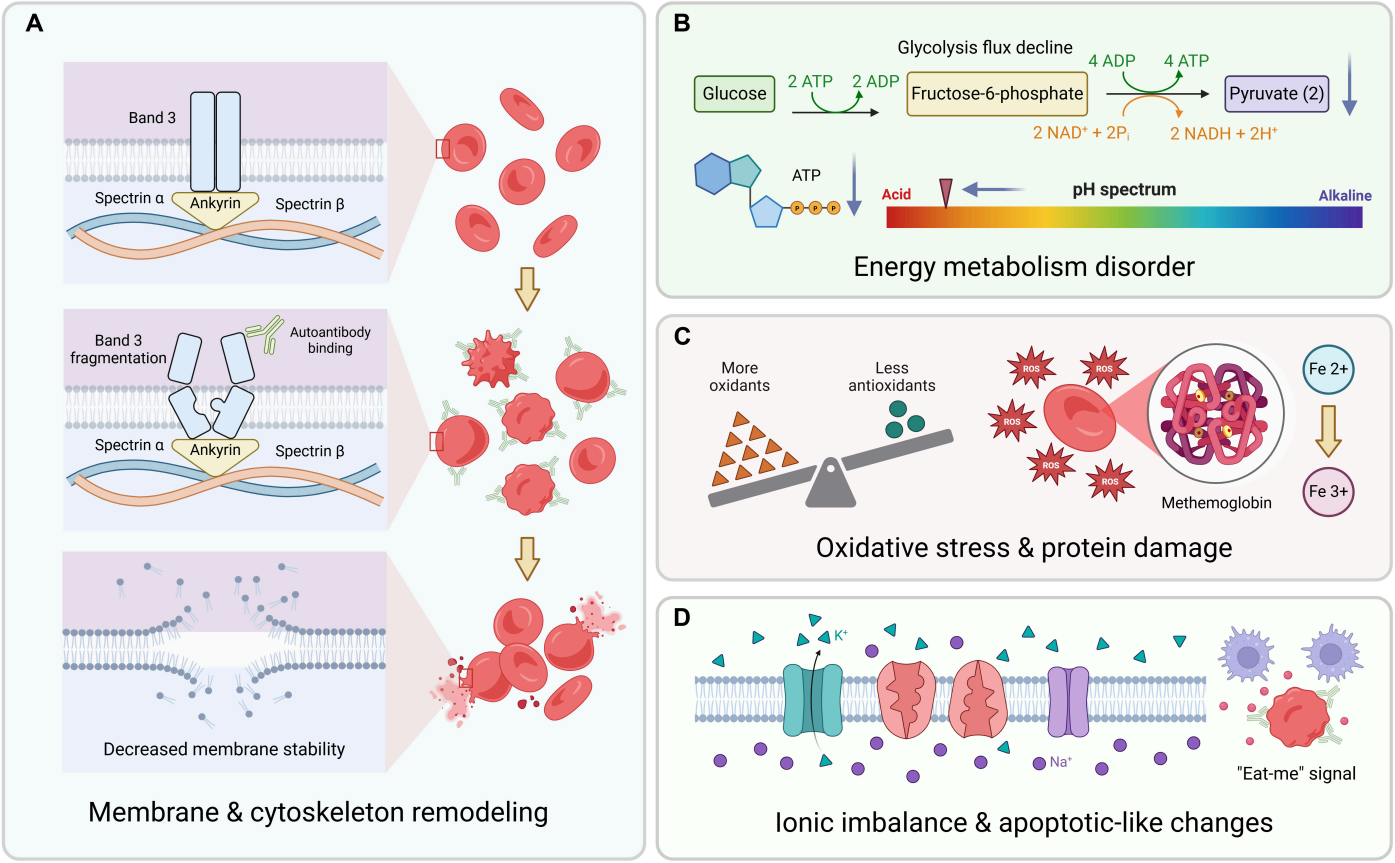
Key mechanisms underlying RSL. (A) Membrane and cytoskeleton remodeling involves structural damage of spectrin ankyrin and band 3 protein complex. (B) Energy metabolism disorder presents declined glycolysis flux ATP depletion and pH reduction in stored red blood cells. (C) Oxidative stress and protein damage cause ROS accumulation, methemoglobin formation, and decreased membrane stability. (D) Ionic homeostasis disturbance and apoptosis-like changes lead to calcium overload phosphatidylserine externalization and “eat-me” signal exposure. Created in BioRender. Lei, Y. (2026) https://BioRender.com/ru4zlae.

Band 3 protein undergoes aggregation and proteolytic fragmentation under conditions of oxidative stress and altered phosphorylation status, generating novel conformational epitopes that facilitate binding to denatured hemoglobin and autoantibodies. These molecular events not only drive microvesiculation but also establish the structural prerequisite for subsequent immune recognition and macrophage-mediated clearance [14]. Such remodeling of membrane proteins and the cytoskeleton ultimately manifests as impaired RBC deformability and increased osmotic fragility, which can be quantified through various in vitro indices including ektacytometry, density gradient fractionation, and morphological scoring [11] (Fig. 2A).

#### Energy metabolism impairment

Mature RBCs rely exclusively on glycolysis for ATP generation. During storage, progressive decline in glycolytic flux leads to sequential depletion of ATP and 2,3-DPG during early and mid-to-late storage phases, respectively, representing a central metabolic characteristic of RSL [3].

Under standard blood bank conditions, gradual glucose consumption coupled with intrabag lactate accumulation induces pH reduction, thereby inhibiting glycolytic and associated metabolic enzyme activities and exacerbating energetic insufficiency [15]. ATP depletion is intimately linked to impaired function of transmembrane pumps such as Na^+^/K^+^-ATPase (adenosine triphosphatase), resulting in intracellular potassium efflux, sodium influx, and disruption of cation homeostasis. These ionic perturbations coincide with morphological transitions of RBCs from biconcave discs to echinocytes and spherocytes, accompanied by increased microvesiculation [16].

Large-scale metabolomic and genetic analyses of donor cohorts further demonstrate that polymorphisms in genomic regions encoding key glycolytic enzymes—including phosphofructokinase platelet (PFKP) and hexokinase 1 (HK1)—markedly influence end-of-storage ATP and hypoxanthine levels. These metabolic phenotypes correlate with the degree of hemolysis both in vitro and in vivo, indicating that the severity of energy metabolism impairment is donor-specific and may affect post-transfusion outcomes [17] (Fig. 2B).

#### Oxidative stress and hemoglobin/cytoskeletal damage

Omics and biochemical investigations consistently demonstrate that RBCs undergo progressive oxidative deterioration during cold storage, characterized by escalating reactive oxygen species (ROS) accumulation, lipid peroxidation, and protein carbonylation, concurrent with the gradual exhaustion of antioxidant defenses including glutathione and nicotinamide adenine dinucleotide phosphate (NADPH) [3].

In long-stored RBCs, substantial quantities of oxidatively modified hemoglobin species—notably methemoglobin and hemichromes—accumulate and form covalent, nonreducible crosslinks with cytoskeletal components, generating hemoglobin-spectrin crosslinked adducts that compromise membrane mechanical stability and promote microvesiculation [13]. Oxidative injury further induces extensive posttranslational modifications of membrane proteins, particularly evident as aggregation, proteolytic fragmentation, and altered phosphorylation status within the band 3 (AE1) complex, changes that align with the observed restructuring of band 3-centered interactomes [18].

Time-course correlation analyses reveal that elevated levels of glutathione precursors, extracellular antioxidant capacity, and proteasomal activity during early storage phases negatively predict subsequent lipid peroxidation and hemolytic indices, indicating that oxidative stress coupled with impaired protein quality control constitutes a principal mechanistic driver underlying RSL progression [19] (Fig. 2C).

#### Ionic homeostasis disturbance and “apoptosis-like” changes

With prolonged storage, RBCs gradually lose the Na^+^/K^+^ gradient, and the potassium (K^+^) concentration in the storage bag rises in an approximately linear manner. Meanwhile, increased transmembrane influx of calcium (Ca^2+^) further drives intracellular signaling imbalance and morphological alterations [20].

Elevated Ca^2+^ load is closely associated with phosphatidylserine (PS) externalization, membrane blebbing, and microvesicle release, exposing more negatively charged phospholipids on the RBC surface and creating procoagulant surfaces for the assembly of thrombin complexes [20]. In both in vitro and in vivo aging models, band 3 clustering, spectrin degradation, PS externalization, and conformational changes in CD47 collectively constitute “eat-me” signals, which are recognized by multiple macrophage receptors and autoantibodies. Similar molecular markers emerge progressively during storage and enhance the propensity for RBC clearance [12].

A systematic review of “aging and death signaling” in mature RBCs demonstrates that storage-induced oxidative damage, membrane-skeleton remodeling, and phospholipid distribution disorder overlap extensively with physiological RBC aging across multiple pathways, thereby linking the RSL to accelerated “eryptosis-like” or “senescence-like” clearance processes [21] (Fig. 2D).

### Clinical harms of RSL

Following transfusion of RBCs stored under standard blood bank conditions, approximately 5% to 10% of the cells are cleared within 24 h; for RBC concentrates nearing their expiration date, this early clearance rate can rise to more than one quarter, directly reducing the effective RBC dose provided per unit transfusion [11].

Autologous transfusion studies in healthy volunteers demonstrated that transfusion of RBCs after 1 to 6 weeks of refrigerated storage resulted in a significant linear increase in serum indirect bilirubin and ferric iron levels, directly reflecting that extravascular hemolysis of storage-lesioned RBCs (mediated by the hepatic and splenic mononuclear phagocyte system) is exacerbated with prolonged storage. Notably, 78% of subjects in the 6-week storage group developed circulating non-transferrin-bound iron that persisted for ≥10 h, indicating that the rate of iron release from RBCs at this storage duration exceeded the binding capacity of transferrin, overwhelming the physiological capacity of systemic iron metabolism and increasing iron burden release [22].

Murine model studies further confirmed that transfusion of long-stored RBCs induces the production of non-transferrin-bound iron and exacerbates inflammatory responses and adverse outcomes when combined with “second hits” such as infection, suggesting a potential role for storage-related iron overload in increasing susceptibility to infection and aggravating organ injury [23].

The accumulation of free hemoglobin and RBC-derived microvesicles in storage supernatants can rapidly scavenge intravascular NO, thereby inducing vasoconstriction and elevated pulmonary circulatory resistance, which is associated with pulmonary hypertension and microcirculatory dysfunction in multiple animal models [24]. Free hemoglobin and free heme can be filtered into renal tubules and deposited, mediating oxidative stress, cast formation, and renal tubular epithelial injury, both of which are linked to an increased risk of renal function deterioration in clinical and experimental studies, particularly prominent in multiple or high-volume transfusions [16].

Furthermore, PS-enriched microvesicles released by stored RBCs, as well as RBCs themselves with externalized PS, can provide a highly procoagulant membrane surface for thrombin generation, potentially promoting the formation of thrombin–fibrin networks in vivo and exacerbating thrombotic tendency [21].

The concentration of potassium ions in the supernatant of long-stored RBCs is markedly elevated. In isolated rat heart models and human induced pluripotent stem cell (iPSC)-derived cardiomyocyte models, exposure to supernatant from aged RBCs results in bradycardia, prolonged sinus node recovery time, and slowed atrioventricular conduction, indicating that storage-associated hyperkalemia has the potential to directly induce arrhythmias [25].

Studies based on human pulmonary artery endothelial cells demonstrate that, compared with fresh RBCs, long-stored RBCs suppress HIF-1α homeostasis, impair mitochondrial respiratory function, shift endothelial cell metabolism from oxidative phosphorylation to glycolysis, and reduce NADPH and ATP levels, revealing that storage lesions may disturb pulmonary vascular oxygen sensing and energy metabolic homeostasis from an organ-specific perspective [18].

Retrospective analyses of cardiac surgery and pediatric patients suggest that the use of RBCs with longer storage duration (e.g., exceeding 4 weeks or 25 to 38 d) in some high-risk populations is associated with adverse outcomes such as an increased postoperative infection rate, indicating that storage lesions may have a dose- and population-dependent relationship with prognosis in specific clinical settings [26].

### Detection challenges and technical requirements

Although traditional blood bank management uses storage duration as a key reference for RBC quality, large randomized controlled trials have not observed differences in all-cause mortality or multiple organ dysfunction at the population level when comparing fresher versus standard-issue leukoreduced RBC concentrates, indicating that storage time is a crude surrogate rather than a reliable biological phenotypic marker for RSL [2].

Additionally, the extensive existing experimental data on storage conditions and lesion severity primarily focus on the characterization of in-bag metabolism, oxidative stress, and membrane structural alterations, with insufficient quantitative mapping between these in vitro test results and 24-h post-transfusion recovery, organ function changes, and long-term clinical outcomes, which limits the translation of detection indicators into clinical risk stratification tools [27].

Current routine quality control in blood banks still heavily relies on global biochemical parameters such as hemolysis rate, in-bag potassium concentration, and partial RBC indices. Such average indicators fail to reflect the heterogeneity within the cell population and cannot promptly identify small but biologically severely damaged RBC subpopulations [16].

In contrast, experimental methods for assessing morphology, deformability, microvesicle burden, PS externalization, and multi-omics characteristics often require specialized equipment and cumbersome operations, with limited throughput and mostly being endpoint or destructive assays, making them unsuitable for routine screening of every unit of RBCs in large-scale blood supply systems [11].

Although metabolomics and proteomics have uncovered multi-pathway remodeling in RSL, these methods are associated with high costs, long analytical cycles, and complex data interpretation, and are currently mainly used in research settings, failing to meet the demand for real-time or near-real-time quality monitoring in blood banks [3].

Against this backdrop, achieving highly sensitive, multi-parameter detection of RSL with single-cell or subpopulation-resolution while ensuring throughput and operational feasibility, and tightly coupling these detection results with post-transfusion function and clinical outcomes, has become a critical technical bottleneck driving the transition of RSL assessment from research to precise clinical application [28].

## Overview of Microfluidic Chip Platforms

As an emerging analytical and biomimetic system integrating multidisciplinary technologies, microfluidic chip platforms break the limitations of traditional experiments and models through micrometer-scale fluid manipulation and the integration of functional modules [29]. With microchannel networks based on precise hydrodynamic design, compatible material systems, and flexible fabrication processes, microfluidic chips enable high-throughput, low-consumption analysis of biological samples and reconstruct the in vivo physiological microenvironment, supporting diverse scenarios ranging from basic research to clinical applications [30].

From the perspective of technological evolution and application expansion, microfluidic chip platforms have followed a trajectory of increasing biological complexity, from lab-on-a-chip (LOC) to organ-on-a-chip (OOC). LOC, as the basic form, realizes the miniaturization and integration of traditional laboratory functions. In recent years, this has advanced to OOC, which reproduces the physiological and functional characteristics of organs through biomimetic structural design and multicellular system integration [31]. Together, the core concepts, design logics, and technological correlations of the 2 platforms underpin the application of microfluidic technology in relevant research [30,31].

### LOC and OOC: Concepts and relationships

Microfluidic technology manipulates trace fluids within enclosed microscale channels, integrating fluid dynamics, chemical reactions, and biological processes into a single device to achieve high-throughput, low-reagent-consumption, and fast-response analysis and manipulation [32,33]. LOC is generally defined as a miniaturized analytical system that integrates multiple unit operations—including sample pretreatment, reagent manipulation, separation, reaction, and detection—onto a single microfluidic chip. Its core objective is to accomplish “sample-to-answer” integrated detection from raw samples to analytical results within a confined volume [34]. In such systems, modules including sampling, mixing, reaction, and signal readout are arranged on the same substrate to form a compact fluidic and reaction network, thereby reducing analysis time and enhancing automation [35]. LOC platforms can perform quantitative or qualitative detection of diverse targets such as nucleic acids, proteins, and cells within a limited space, providing miniaturized and integrated solutions for in vitro diagnostics and on-site testing.

Building on the general microfluidic integration framework provided by LOC, OOC further reconstructs tissue or organ microenvironments with functional phenotypes and, in certain models, specific anatomical structures, inside the chip [36–39]. By precisely regulating parameters such as shear stress, solute gradients, and extracellular matrix (ECM), OOC simulates in vivo physiological or pathological processes at the microscale [30,40]. OOC typically adopts a multi-chamber or multi-channel layout combined with porous membranes and hydrogel scaffolds to enable substance exchange and signal transduction between different cell populations. It has been used to reproduce the structure–function units of a wide range of organs, including the lung, liver, intestine, and blood vessels [41,42].

From a technical lineage perspective, LOC offers the foundation for fluid manipulation and modular integration based on microchannel networks, whereas OOC superimposes requirements such as multicellular coculture, long-term perfusion, and biomimetic mechanical stimulation on this basis, representing a highly specialized and biomimetic extension of LOC in the fields of biomedicine and tissue engineering [43]. At the systematic methodological level, OOC is classified as a microphysiological system formed by the intersection of microfluidic systems and tissue engineering. Its fluid control and material selection directly inherit the engineering foundation of LOC while undergoing deeper customization around specific organ functions in terms of structure and operating conditions [44,45].

### Structural design of microfluidic chips

#### Design of LOC

##### Unit operation modules and channel topology

A LOC consists fundamentally of inlets for sample and reagent introduction, multi-segment functional microchannels and chambers, and integrated detection zones. Geometric interconnection and fluidic resistance design between individual units form a fluidic network with specific spatiotemporal sequences [34]. In a typical structure, a sample enters the pretreatment region via the inlet, where operations such as filtration, enrichment, or lysis are completed before being delivered to mixing and reaction chambers. Target analytes are then separated and detected through separation channels or detection chambers, enabling a full analytical workflow within a closed microenvironment [35]. Diagnostic LOCs often sequentially couple steps including nucleic acid amplification, immunoreaction, and signal amplification within microchannel networks to reduce manual operation and improve sensitivity and the reproducibility of results [34].

##### Driving modes and structural types

In LOC systems, driving mode and structural format determine flow stability, controllability, integration strategy, reagent consumption, and the division of functions between the disposable chip and the external instrument, making them more informative classification axes than application labels alone [46–48]. Pressure-driven continuous-flow platforms, centrifugal microfluidics, electrokinetic systems, droplet microfluidics, and digital microfluidics therefore represent distinct engineering solutions rather than interchangeable implementations of the same concept [46,48,49].

Pressure-driven continuous-flow LOCs remain the most general architecture for assays that require sustained perfusion, defined residence time, and quantitative control of flow velocity and shear stress [46,48,50]. Their main advantage is the ability to support stable, nonpulsatile transport over a broad operational range with straightforward coupling to channel networks, mixers, separators, and downstream detectors [48,50]. This format is also well suited to multistep analytical workflows because continuous injection and collection facilitate real-time monitoring and sequential processing [49]. However, these systems usually rely on external syringe, peristaltic, or pneumatic hardware, which increases instrument footprint, tubing dead volume, bubble sensitivity, and system-level complexity [50,51]. Reproducibility is therefore often limited less by chip geometry than by backpressure, tubing compliance, connector quality, and incomplete reporting of operating conditions, all of which can shift the actual on-chip hydrodynamic environment away from the nominal pump setting [51,52]. From a translational perspective, pressure-driven LOCs are technically mature but less attractive for portable or low-infrastructure deployment unless peripheral hardware is substantially simplified [47,50].

Centrifugal microfluidics addresses integration more directly by using rotationally generated body forces to move liquids through radially distributed chambers and channels, enabling transport, metering, mixing, aliquoting, switching, and valving on a single disc [53,54]. Because propulsion is generated without external fluidic interfaces, the format supports closed cartridges, pulse-free liquid handling, and straightforward multiplexing across many assay units on the same substrate [53,55]. Processed volumes are readily scalable from nanoliters to milliliters, which broadens compatibility with clinical chemistry, immunoassays, blood handling, and nucleic acid workflows [53]. This high level of unit operation integration makes centrifugal platforms one of the most clinically advanced LOC formats for sample-to-answer diagnostics [53,55]. Their limitations arise from the need for rotational instrumentation, the difficulty of implementing certain flow patterns within a spinning geometry, and the sensitivity of burst-valve behavior to fabrication tolerances and surface finish [53,55,56]. Clinical readiness is therefore strong at the platform level, but further progress still depends on manufacturable valving, environmental robustness, and cost-effective deployment hardware [55].

Electrokinetic microfluidics, including electroosmotic pumping, offers a different balance of advantages by generating pulse-free flow without moving mechanical parts and by integrating naturally with planar microfabrication [57,58]. In these systems, fluid transport is controlled electrically rather than mechanically, which enables compact architectures, low reagent consumption, and precise manipulation in analytical chips [50,57,58]. Electrokinetic methods are particularly attractive where miniaturization, integration density, and accurate low-volume handling are more important than high volumetric throughput [50,57]. Their main limitations follow directly from the driving mechanism: Performance depends strongly on surface charge, ionic strength, pH, and electric field strength, so flow can drift when channel chemistry or sample composition changes [58]. High-voltage requirements, electrode electrolysis, gas formation, local pH shifts, and material degradation further complicate robust operation, especially in assays involving complex biological matrices [57,58]. Reproducibility can therefore be high under well-controlled analytical conditions but poorer in heterogeneous real-world samples, which has slowed broad clinical deployment relative to more mechanically robust LOC formats [50,58].

Droplet microfluidics replaces continuous single-phase streams with discrete compartments in an immiscible carrier phase, thereby reducing Taylor dispersion, suppressing wall-mediated cross-contamination, and accelerating mixing and mass transfer at very small volumes [59]. This architecture is especially powerful for ultrahigh-throughput screening, reagent economy, single-cell encapsulation, and statistically rich assays in which each droplet functions as an isolated microreactor [59,60]. Droplets can be generated at very high frequencies, and operations such as merging, splitting, spacing, sorting, and pico-injection provide a versatile liquid-handling toolkit at picoliter-to-nanoliter scale [59,61]. The main limitation is that high-performance droplet workflows often depend on complex peripheral infrastructure for droplet generation, synchronization, incubation, reinjection, and coupling to downstream detectors or off-chip analytics [59,62]. Translation is further constrained by the difficulty of interfacing oil-phase droplets with external workflows and by the gap between proof-of-concept manipulation and robust end-to-end assay automation [59,61]. Reproducibility can be excellent when droplet size and frequency are tightly controlled, but passive droplet systems remain sensitive to surface chemistry and ambient fluctuations, whereas active control improves consistency at the cost of throughput and setup simplicity [61,62].

Digital microfluidics also operates on discrete droplets, but instead of relying on immiscible-flow compartmentalization, it manipulates droplets directly on electrode arrays, most commonly through electrowetting-on-dielectric actuation [63–65]. The core strength of this format is programmability: Transport, dispensing, merging, splitting, and mixing are executed through software-defined addressing, allowing multiple assay sequences to be reconfigured on the same planar chip [64–66]. This reconfigurability, combined with low reagent consumption and compatibility with integrated sensing, has made digital microfluidics attractive for nucleic acid testing, immunoassays, blood analysis, and other multistep LOC applications [65–67]. Relative to channel-based continuous-flow systems, digital microfluidics offers greater protocol flexibility and simpler logical reprogramming of liquid-handling steps [63,65]. Its limitations are primarily interfacial and materials-based: Protein and lipid fouling can degrade droplet mobility, evaporation can alter concentration, routing becomes more difficult as parallel assay density increases, and volumetric throughput remains modest [65,68]. Clinical translation has progressed in portable assay formats, but broader deployment still requires more durable surfaces, reliable sample-loading interfaces, and tighter integration between disposable fluidic layers and reusable control electronics [65–67].

Taken together, these platforms occupy different positions in the LOC design space rather than forming a simple hierarchy of advancement [46,48]. Pressure-driven systems remain the most versatile choice for general continuous-flow manipulation and quantitative hydrodynamic control [48,50]. Centrifugal platforms offer the strongest route to compact workflow integration and sample-to-answer automation in diagnostic LOCs [53,55]. Electrokinetic methods are advantageous when compactness, pulse-free flow, and planar integration are prioritized, but they remain constrained by electrochemical and buffer-dependent instability [57,58]. Droplet microfluidics is unmatched for throughput and compartmentalized microscale reactions, yet its real-world adoption is still limited by interfacing and peripheral complexity [59,61]. Digital microfluidics provides the highest degree of software-level liquid-handling programmability, but its broader clinical readiness depends on solving fouling, evaporation, durability, and workflow-integration bottlenecks [65–67]. Accordingly, LOC platform selection should be based on the required balance among flow control, integration density, assay programmability, reproducibility, and translational robustness rather than on microfluidic miniaturization alone [46–48].

#### Design and construction of OOC

##### Tissue interface and spatial compartmentalization

By employing multi-chamber or multi-layer microchannel configurations, OOC platforms establish spatially separated yet mass-permeable tissue interfaces, allowing fluidic networks to fulfill not only substance transport tasks but also the reconstruction of physiological microenvironments [40,69]. In models of blood–tissue or epithelial–endothelial interfaces, porous polymeric membranes are commonly embedded between upper and lower flow channels, allowing distinct cell types to be seeded in the upper and lower chambers, respectively. This reconstructs barrier functions and transmembrane transport properties, simulating in vivo tissue interfaces at the microscale [40]. By perfusing culture medium or blood-mimicking fluid separately in different chambers, OOC can maintain channel connectivity while providing differentiated chemical and mechanical environments for diverse cell populations, achieving independent regulation of different microenvironments [41].

##### Mechanical microenvironment and long-term perfusion

In terms of fluidic design, OOC can apply continuous perfusion in one channel to simulate blood flow-induced shear stress while maintaining a static or low-shear environment in the adjacent channel. This mirrors the mechanical disparity between tissue-facing and blood-facing surfaces in vivo, realizing precise regulation of shear stress and solute gradients exerted on cells [41].

Unlike most OOCs dominated by epithelial–mesenchymal structures, endothelium-on-a-chip (EOC) impose higher requirements on perfusion conditions. Stable and controllable intraluminal perfusion and trans-wall pressure gradients must be maintained throughout the culture period, thereby simultaneously driving shear responses, convective-diffusive transport, and hemorheological behaviors [70]. Within the design framework of vascularized tissue construction, flow velocity and channel dimensions not only determine shear stress and pressure drop but also directly influence oxygen and nutrient diffusion distances. Consequently, EOC must simultaneously satisfy the dual constraints of mechanics and mass transport—a balance that is not prominent in many static culture-dominant chips [71,72].

Based on the above characteristics, the design of EOC typically targets the geometric and hemodynamic parameters of specific vascular beds. By adjusting channel diameter, length, and branching topology, wall shear stress and residence time close to the in vivo scenario are reconstituted at a given volumetric flow rate [71]. Under laminar flow conditions, the shear stress can be estimated from the analytical relationship between channel radius and flow rate. EOC mimicking microvessels mostly constrain shear stress within the range of 1 to 10 dyn/cm^2^ to match the physiological shear load of the microcirculation while avoiding endothelial detachment [73].

In prepatterned vascularized devices, tailored ranges of wall shear stress and pressure gradients can be achieved at distinct locations by adjusting channel cross-sections and branching resistances. This enables the simulation of different flow environments, such as those found in arteries, venules, or sinusoidal vessels [74]. For instance, the AngioChip achieves similar shear levels across all branches by designing primary and secondary branching channels as 50 × 200 μm and 50 × 100 μm, respectively, and balancing hydraulic resistance in the network layout, thereby facilitating uniform mechanical stimulation throughout the entire network [75]. Hemodynamics-oriented design practices demonstrate that modeling pathological vascular environments requires the generation of spatially nonuniform shear and recirculation zones within the same chip. This is achieved through stenotic, curved, or bifurcated geometries, which recapitulates the local stress characteristics at predilection sites for atherosclerosis and aneurysms [76].

In addition to regulating shear stress, biomimicry of the mechanical microenvironment also encompasses the simulation of cyclic strain. Some OOCs are equipped with deformable chambers, and periodic deformation of thin walls can be achieved via negative-pressure traction or mechanical loading, thereby applying in vivo-mimetic cyclic strain at the microscale. This design could simulate alveolar respiratory movements or vascular pulsations, extending static channels in LOCs into biomimetic systems with dynamic mechanical dimensions [40].

For long-term culture scenarios, OOCs require structurally reserved pathways for culture medium replacement and gas exchange to support stable perfusion and cell function maintenance over days or even weeks. This demand has driven the systematic optimization of chamber volume, pathway layout, and connection modes based on LOC channel networks [43].

##### Blood cell manipulation geometries

In the design of blood-related structures, a variety of passive separation geometries developed in LOC systems provide referable basic units for the upstream blood cell processing modules of OOC platforms. For instance, size- and hydrodynamics-based structures such as branched channels, step structures, and pillar arrays are employed to achieve physical sorting and enrichment of RBCs and other blood cells [77]. In such passive blood cell separation chips, the cross-sectional shape of channels and array layout directly determine the trajectories of different blood cells in the flow field, thereby affecting the separation efficiency and shear exposure of RBCs and other cell populations. This lays a geometric design foundation for the precise control of blood cell states entering organ models in subsequent OOC systems [77].

In cell and microbead sorting devices, mechanisms including filtration, hydrodynamic focusing, dielectrophoresis, acoustophoresis, and magnetophoresis are utilized to design sorting modules with specific resolution and throughput. These unit operations can either serve as independent analytical modules in LOCs or be integrated upstream or downstream of OOCs to selectively control cell populations involved in organ models [78].

In studies on the blood–endothelium interface, EOCs are required to carry intact blood components and maintain stable flow fields under non-Newtonian rheology and cell–cell interaction conditions, which substantially exceed the general requirements of many OOC systems that only use culture media or cell-free fluids [79]. When the size of RBCs is comparable to the diameter of microvessels, the Fåhræus–Lindqvist effect and cell margination occur within the chip, leading to alterations in local viscosity and wall shear stress distribution. Such hemorheological effects are regarded as key physical characteristics that distinguish EOC from other fluidic chips [73].

### Common materials and their selection criteria

#### Material systems for LOC

##### Inorganic substrates

In LOC devices, glass and silicon substrates serve as a foundation for fabricating high-precision microchannel networks by leveraging mature microelectronic manufacturing processes. High-aspect-ratio and highly reproducible channel geometries can be achieved via photolithography and etching, making them suitable for analytical systems sensitive to fluidic resistance and mass transfer processes [80].

Glass exhibits excellent optical transparency in the visible and near-ultraviolet (UV) bands with low autofluorescence, providing a high-quality optical path for on-chip fluorescence and absorption spectroscopy detection and facilitating the integration of diverse optical readout modules on LOCs [80].

In terms of chemical stability, glass and silicon demonstrate strong resistance to most aqueous systems and some organic solvents, adapting to multi-step analytical workflows requiring organic-phase extraction or complex buffer systems, and offering stable interfaces for integrated chemical and biological detection [81].

##### Elastomeric materials

Elastomeric materials, typified by polydimethylsiloxane (PDMS), are extensively used in LOC prototype development, owing to their low cost, facile moldability, and excellent optical transparency [72,82]. PDMS precursors can be cross-linked and cured at room or moderate temperatures without high-pressure or high-temperature equipment, enabling researchers to complete the full process chain from mold replication to chip packaging in conventional biological laboratories, thereby lowering the threshold for iterative LOC design [82].

After oxygen plasma treatment, PDMS layers can form irreversible bonds with glass or another PDMS layer to create sealed microchannel networks. Electrodes or flexible membranes can be pre-embedded during molding to construct microvalves, micropumps, and electrochemical detection units, realizing the integration of fluid manipulation and signal acquisition [82].

In cell-related LOC applications, the gas permeability of PDMS facilitates the diffusion of oxygen and carbon dioxide through the chip walls, improving gas exchange in the local culture environment. However, the marked adsorption and permeation of hydrophobic small molecules and drugs may alter solute concentrations in channels over time, posing challenges to the accuracy of quantitative analysis and drug efficacy evaluation [83]. Rapid water evaporation occurs in open PDMS microfluidic systems, which may cause solute concentration and osmotic pressure changes in small-volume channels; this effect must be mitigated through packaging and environmental control in diagnostic chips involving high-concentration protein solutions or blood samples [83].

##### Thermoplastic polymers

Thermoplastic polymers including polymethyl methacrylate (PMMA), polycarbonate (PC), polystyrene, and cyclic olefin copolymers (COC) enable high-fidelity replication of microchannel structures via hot embossing and injection molding. They support the mass manufacturing of disposable diagnostic cartridges at low material costs and with high mechanical strength, facilitating the transition of LOCs from laboratory prototypes to clinical product formats [80].

Such polymeric materials have been widely adopted in conventional cell cultureware and diagnostic consumables. Their fundamental biocompatibility and mature manufacturing processes lay a foundation for their application in LOCs, ensuring good compatibility between chip architectures and existing laboratory infrastructure [82].

Regarding surface properties, plasma or UV/ozone treatment can substantially enhance the hydrophilicity of polymer surfaces, improving channel wettability and reducing bubble formation to guarantee stable continuous-flow operation [82]. Further modification through grafting hydrophilic polymers or coating protein-repellent layers can reduce nonspecific protein adsorption, thereby improving analyte recovery and signal-to-noise ratios in immunoassays and nucleic acid detection [82]. For whole-blood sample analysis, the introduction of anti-protein and anticoagulant coatings on polymer surfaces helps suppress plasma protein deposition and platelet adhesion, enhancing the hemocompatibility of microchannels and lowering clogging risks, which makes LOCs more suitable for handling complex blood samples [84].

#### Material extension from LOC to OOC

##### PDMS system optimization

OOC predominantly adopts the well-established PDMS system from LOC to ensure the fabrication precision of microchannels and meet the requirements for optical imaging. Meanwhile, additional constraints and modifications are imposed on this material due to the demands of long-term culture, biomimetic mechanical stimulation, and blood contact [43].

PDMS exhibits excellent gas permeability, which can provide sufficient oxygen supply for high-density cell culture and the construction of thick-layer tissues in microscale structures, enabling it to support more complex 3-dimensional (3D) physiological systems based on the traditional LOC channel architecture [40]. Furthermore, the favorable flexibility and elasticity of PDMS allow for the construction of vacuum chambers or elastic support structures outside the chip. Periodic negative pressure drives the controllable deformation of the membrane structure and channel sidewalls, thereby applying cyclic mechanical strain within the physiological range to cell layers and simulating organ-specific mechanical microenvironments such as alveolar respiration and vascular pulsation. This mechanical design dimension is generally not a core consideration for traditional diagnostic LOC [40].

Nevertheless, under long-term in vitro culture conditions, the nonspecific adsorption of hydrophobic small molecules and signaling molecules by PDMS alters the local concentration distribution of substances, further compromising the accuracy of drug efficacy and toxicity evaluations. Therefore, while inheriting the mature fabrication processes of LOC, OOC design requires more rigorous quantitative characterization and regulation of the material's adsorption behavior [83].

To address the aforementioned issues, current studies primarily adopt strategies such as elastomer material substitution, surface modification coating, and composite substrate construction to reduce small-molecule adsorption and enhance the chemical stability of materials [85]. Additionally, some studies have utilized stretchable cellulose ester films to replace traditional PDMS membranes, which markedly suppresses small-molecule adsorption while retaining favorable optical properties and mechanical elasticity [86].

##### Rigid substrates

To enhance the mechanical stability of tissue-mimetic systems and facilitate scalable manufacturing, OOC platforms have adopted glass and thermoplastic polymers—well-established materials in LOC devices—as substrates and channel layer materials. These rigid materials enable the fabrication of microchannels and chambers with higher pressure resistance and reduced deformation, thereby supporting continuous perfusion for days to weeks [80].

In OOC systems, glass substrates not only offer excellent optical transparency and chemical stability but also provide a robust window for high-resolution real-time imaging and multiple rounds of medium replacement, enabling the integration of more sophisticated biomimetic structures onto conventional LOC detection platforms [80].

Thermoplastic polymers can be mass-produced with complex microchannel architectures via validated LOC fabrication techniques such as hot embossing or injection molding, aligning OOC material and manufacturing pathways with the industrial chain of diagnostic LOC devices. However, under long-term culture and repeated sterilization conditions, greater attention must be paid to the impacts of material leaching and surface aging on the cellular microenvironment [82].

In multi-chamber OOC configurations, these rigid materials typically serve as supporting layers to carry flexible layers (e.g., PDMS or hydrogels). Interlayer bonding constructs multilayer microfluidic systems with well-defined interfaces and stable geometries, enabling structural reinforcement of biomimetic tissue interfaces based on traditional LOC channel networks [43].

##### Porous membranes and hydrogels

Building on the planar channel-dominated material system of LOC devices, OOC platforms further incorporate tissue engineering materials such as porous polymer membranes and cross-linkable hydrogels to reconstruct tissue interfaces with selective permeability and 3D ECM environments. These materials are rarely used or only serve as supplementary components in conventional analytical LOC devices [43,87].

Porous polymer membranes (e.g., polycarbonate) act as physical barriers at blood–tissue interfaces in epithelial–endothelial coculture models. As a classic research model of biological barriers, the blood–brain barrier (BBB) is an essential structure that maintains homeostasis within the central nervous system [88,89]. The pore size and thickness of these membranes determine the transmembrane migration of small molecules, proteins, and cells between separated chambers, which directly controls barrier integrity and substance transport efficiency—rendering such membranes irreplaceable for in vitro BBB models [40,90].

Cross-linkable hydrogels are injected into designated chambers and cross-linked in situ to form 3D ECM scaffolds with tunable elastic modulus and porous architecture, which helps maintain the functional phenotypes of parenchymal cells (e.g., hepatocytes and cardiomyocytes). This endows OOC systems with an in vivo-like 3D microenvironment on the basis of LOC fluidic control [43].

In OOC systems involving blood perfusion, hydrophilic or anti-protein adsorption coatings applied to channel and membrane surfaces reduce the adhesion of plasma proteins and platelets, lowering the risk of microthrombosis and mechanical damage to RBCs. This enables controllable regulation of blood–material interactions in LOC-derived organ systems [84].

##### 3D printing and photosensitive resins

Stereolithography-based 3D printing typically employs resins containing photosensitive functional groups as structural materials, which are used to fabricate complex 3D channels and scaffolds via layer-by-layer photopolymerization, enabling the rapid prototyping of OOC housings, fluidic channels, and supporting structures [91].

For applications in OOC systems, 3D printing materials must satisfy requirements for mechanical strength and resolution while also maintaining optical transparency and biocompatibility; the currently available compatible material systems remain relatively limited [92]. Existing studies have constructed perfusable tubular structures via 3D printing and embedded them within injectable ECM hydrogels to mimic the renal proximal tubule and microvascular interface, demonstrating the application potential of photosensitive resins in the fabrication of 3D microstructures [93].

### Main fabrication methods

#### Typical methods for LOC fabrication

**Fig. 3.**
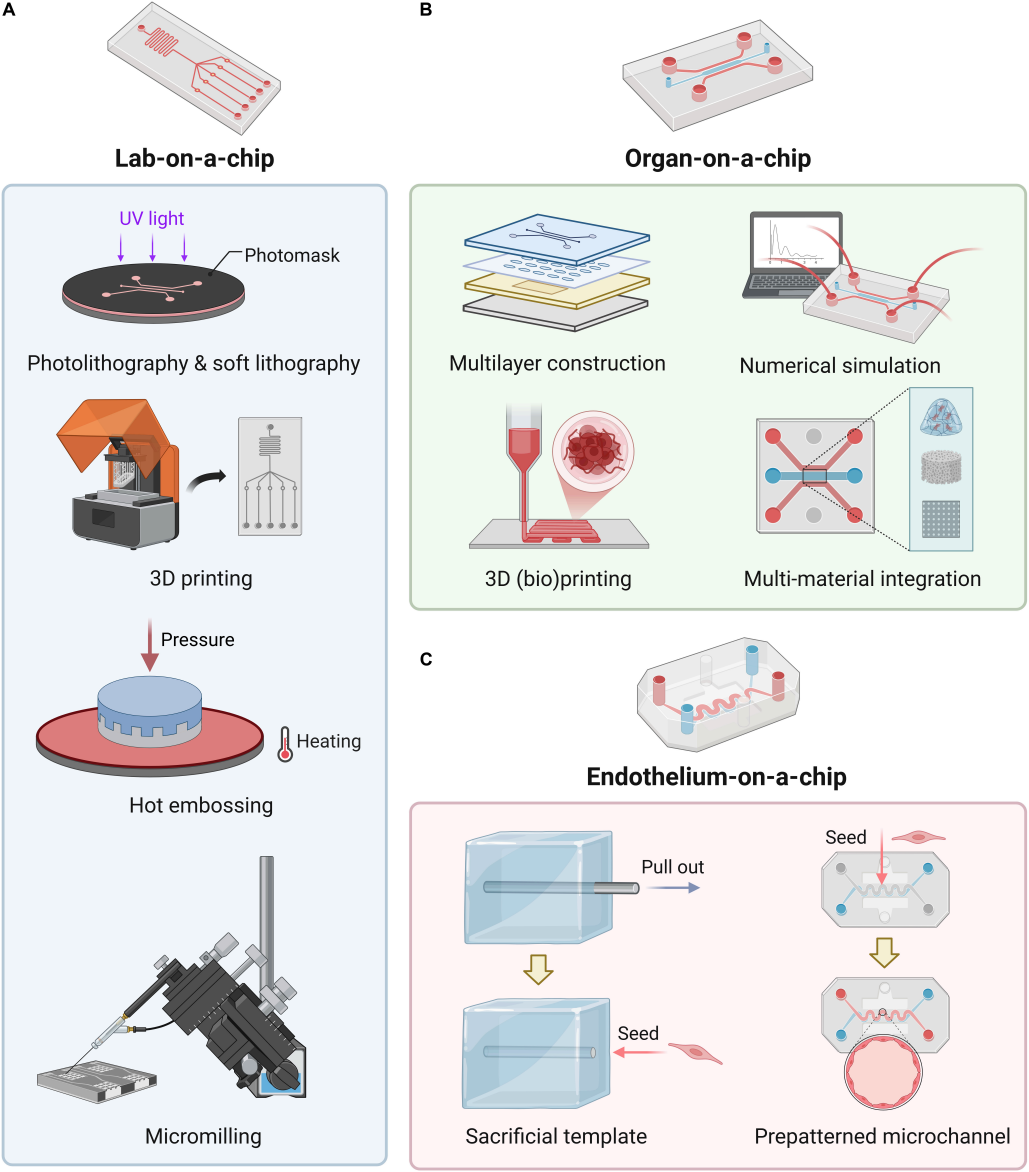
Fabrication technologies for LOC, OOC, and EOC platforms. (A) Core fabrication processes for LOC include photolithography and soft lithography, 3D printing, hot embossing, and micromilling. (B) Advanced manufacturing strategies for OOC encompass multilayer construction, numerical simulation, 3D (bio)printing, and multi-material integration. (C) Specialized fabrication approaches for EOC include the use of sacrificial templates and prepatterned microchannels. Created in BioRender. Lei, Y. (2026) https://BioRender.com/2xbstnb.

The fabrication of LOC devices relies on a suite of mature, scalable microengineering techniques. These methods balance resolution, cost, and throughput to support everything from rapid prototyping to mass clinical manufacturing. Below are the 4 core fabrication routes for LOC platforms (Fig. 3A).

##### Photolithography and soft lithography

In LOC fabrication, photolithography combined with soft lithographic replication represents the core technical route for constructing PDMS-based microchannel networks [94]. This approach enables the production of high-resolution microstructures without the need for expensive mass-production molds, providing a critical process foundation for rapid design iteration [80].

The process typically involves photolithography and etching on silicon or glass substrates to form raised structures of predefined geometry. PDMS precursor is then cast or spin-coated onto the mold surface, cross-linked, cured, and peeled off to yield an elastomeric sheet with negatively replicated channels [82]. PDMS sheets activated by oxygen plasma treatment can be bonded to glass or another PDMS layer to form sealed microchannel networks. Electrodes or flexible membranes can be pre-embedded during fabrication to enable the integration of microvalves, micropumps, and electrochemical detection structures, thereby accomplishing fluid manipulation and signal acquisition within a single LOC platform [82].

In microfluidic chemical analysis and biosensing research, soft lithography is widely employed for rapid iteration from proof-of-concept to functional optimization, laying the microfabrication foundation for the subsequent adoption of analogous channel, valve, and pump structures in OOC systems [95].

##### 3D printing

With the advancement of additive manufacturing technologies, 3D printing has been increasingly incorporated into LOC fabrication workflows to construct complex 3D channels and multilayer structures that are difficult to achieve via conventional planar processing [80].

Stereolithography-based 3D printing can fabricate multilayer intersecting and tortuous microchannel networks in a single process by layer-by-layer exposure and curing of photopolymer resins. This reduces repetitive alignment and bonding steps, enhances structural design flexibility, and offers new geometric space for the construction of complex mixing and separation modules [96].

In microfluidic system studies combined with numerical modeling, 3D printing is frequently used to rapidly validate the effects of different channel layouts and mixing structures on flow field and mass transfer behaviors. This provides geometric design support for subsequent large-scale production using conventional processes [96].

For digital microfluidic platforms, additive manufacturing is also utilized to fabricate modular carriers incorporating electrode arrays and packaging structures. This facilitates the compact integration of droplet manipulation units with optical or electrochemical biosensing modules and offers structural support for highly programmable droplet operation within the LOC system [28,97].

In blood-related microfluidic research, the combination of 3D printing and conventional fabrication processes is regarded as a potential approach for constructing complex pretreatment modules and vascular tree-like networks, providing transplantable structural blueprints for blood flow pathways and microcirculation models in subsequent OOC systems on the LOC architecture [77].

##### Hot embossing

For thermoplastic polymers such as PMMA, PC, and COC, the hot embossing process enables high-fidelity replication of microstructures in medium-volume production, offering a scalable manufacturing pathway for clinically oriented LOC devices [80].

In the hot embossing procedure, target microstructures are first fabricated on a metal or silicon mold. Pressure is then applied at a temperature above the glass transition temperature of the polymer, causing the sheet to soften, flow, and fill the mold cavities. Upon cooling and solidification, demolding yields a chip substrate with precisely defined channel geometries [82].

Compared with soft lithography, hot embossing imposes stricter requirements on mold precision as well as temperature and pressure control. However, within an appropriate process window, it markedly improves interdevice uniformity and mechanical strength, making it suitable for diagnostic chips that demand rigorous control of fluidic resistance and reaction time [81].

Injection molding involves injecting molten polymer into a microstructured mold cavity followed by cooling and solidification. This enables the mass production of highly consistent microfluidic chips under continuous manufacturing conditions, supporting large-scale fabrication of disposable diagnostic cartridges and kits and facilitating the translation of LOC from laboratory settings to clinical and commercial applications [80].

As these large-scale manufacturing processes mature, identical mold and material systems can also be employed for the batch fabrication of rigid support layers and interface modules in OOC devices, achieving integrated layout with LOCs at the manufacturing level [82].

##### Micromilling

In the fabrication of microengineered physiological systems, subtractive manufacturing techniques, such as mechanical machining and cutting, are frequently cited alongside lithography, laser processing, soft lithography, and other methods. Current perspectives suggest that subtractive processes can serve as an effective, low-cost supplementary approach for rapid prototyping within specific material systems and dimensional ranges [91].

From the evolution of early micromachining technologies to emerging additive manufacturing, mechanical cutting processes such as xurography are regarded as viable alternatives for applications with relatively relaxed feature size requirements. Meanwhile, the limitations of such methods in terms of reproducibility and throughput remain to be further investigated and improved [98].

#### Fabrication of OOC structures

OOC fabrication is developed from the mature microfabrication system of LOC and further integrates tissue engineering requirements to form a complete technical route including multilayer construction, 3D bioprinting, multi-material integration, and numerical simulation-aided design. These technologies balance biomimetic fidelity, long-term operational stability, and scalable manufacturing, supporting the reliable reconstruction of organ-level physiological functions (Fig. 3B).

##### Multilayer PDMS OOC

The fabrication process of OOC is largely built on the mature soft lithography and multi-layer bonding technologies of LOC [99]. By replicating microchannels in PDMS and bonding them to glass or polymer substrates, 3D networks comprising multiple perfusion channels and cell culture chambers are constructed, combining the channel integration capability of LOC with the requirements of tissue engineering [43].

Among these, PDMS soft lithography is widely recognized as the standard method for OOC fabrication. Its advantages lie in the mature and stable process, precise realization of micro-scale structures, and excellent compatibility with techniques such as microimaging and flexible actuation [44]. However, this method typically requires photolithographic masks and multi-step processes, the replication step is highly dependent on manual operation, and it is mostly limited to the fabrication of single-sided microstructures, making it difficult to meet the demands of complex 3D structure construction and large-scale production [91]. Therefore, PDMS devices fabricated by soft lithography are more suitable for the early-stage research and development and functional verification of OOC; for the advancement to large-scale applications, integration with other high-throughput molding technologies is still required [93].

In a typical process, the geometries of the upper and lower microchannels and culture chambers are first defined on silicon or glass molds via photolithography. PDMS is then cast and cured to fabricate multilayer elastomeric structures. The layers are subsequently embedded into an integrated device through plasma bonding, enabling the ordered distribution of fluidic and cellular compartments at the microscale. This superimposes vertical tissue architectures onto the channel framework of OOC [82].

In PDMS-based platforms, plasma activation and oxygen plasma treatment not only achieve irreversible bonding between PDMS and glass or thermoplastic substrates but also effectively enhance the hydrophilicity of the inner substrate surfaces, providing favorable conditions for cell seeding and fluid wetting [93].

In organ interface models, porous polymeric membranes are commonly sandwiched between the upper and lower microchannels, allowing separate seeding of epithelial cells and endothelial cells in adjacent chambers to recapitulate tissue barriers and transmembrane transport functions [40,100,101]. This multilayer composite structure, serving as a core structural unit in OOC, is rarely utilized in LOC systems [40]. Such PDMS membrane-incorporated multilayer structures have been employed to construct various organ models including the lung, intestine, and BBB. Precise regulation of shear stress and solute gradients is achieved by perfusing culture medium or blood-mimicking fluid into separate chambers, providing a tunable platform for investigating the behaviors of blood cells within organ microenvironments [40].

In certain OOC devices, the elasticity of PDMS is further utilized to realize controllable mechanical stimulation. Vacuum chambers are placed adjacent to the channels, and cyclic negative pressure is applied to induce bending of the thin membranes and sidewalls, thereby imposing cyclic strains within the physiological range on cell layers. This simulates alveolar respiratory motions or vascular pulsations. Such integrated, mechanically actuated multilayer structures represent a distinguishing feature of OOC from conventional LOC [41].

##### 3D (bio) printing

3D printing accomplishes the formation of 3D structures through layer-by-layer deposition and curing of materials, enabling the direct construction of complex spatial channels and integrated fluidic pathways in a single fabrication process [102]. This confers unprecedented design and manufacturing flexibility for the development of intricate vascular networks, multi-chamber housings, and other structures within OOC systems [91,103].

Within the microfabrication framework of OOCs, representative 3D printing technologies—including stereolithography, material jetting, and extrusion-based printing—all fall under the category of additive manufacturing [104,105]. Their distinct advantages lie in high geometric complexity, elimination of costly mold fabrication, and rapid iterative optimization of design schemes [98]. Nevertheless, 3D printing exhibits certain limitations in terms of processing resolution, surface morphology control, as well as the optical properties and biocompatibility of available materials. Consequently, it is currently predominantly employed for fabricating the macroscopic frameworks or molds of OOCs, whereas the precise construction of the core cellular microenvironment inside the chips still relies on conventional microfabrication approaches such as soft lithography and hydrogel molding [92].

For applications involving living constructs, 3D bioprinting and traditional multi-step microfabrication are regarded as 2 distinct technical routes. 3D bioprinting holds remarkable potential for one-step formation to simultaneously realize tissue architecture and spatial cell arrangement [106–108]. However, its application is constrained by multiple factors, including critical issues such as shear/thermal stress-induced cell damage during the printing process and suboptimal hydrogel crosslinking conditions [109].

Existing studies have fabricated 3D microtissues via 3D bioprinting technology and integrated them with microfluidic channels to construct OOC systems, validating the promising application of this technology in the integrated manufacturing of complex 3D tissues and perfusion interfaces [86]. Furthermore, different 3D bioprinting techniques exhibit distinct compatibility; among them, coaxial printing enables the one-step formation of bilayer tubular structures with an inner endothelial layer and an outer smooth muscle layer, representing the core fabrication technique for artery-like vascular chips [110,111]. Other studies have successfully fabricated multilayered artery-like biomimetic channels using Pluronic F-127 sacrificial ink and coaxial nozzles [112].

In contrast, the 3-step fabrication strategy consisting of mesoscale cavity printing, microvascular sprouting induction, and perfusion mechanical maturation allows the gradual formation of biomimetic microvascular networks with distinct trunk-branch hierarchies in a single chip [113].

In the construction of specific functional models, 3D printing has also been employed to directly form perfusion channels inside hydrogels, thereby enabling the fabrication of biomimetic structures such as the proximal renal tubule–microvascular interface. This demonstrates the important value of combining additive manufacturing technology with ECM engineering in the construction of 3D OOC architectures [93].

##### Multi-material integration

Building on the fabrication basis of LOC systems, which primarily focus on planar channels and simple chambers, OOC systems further adopt multi-material integration processes. They combine materials such as porous membranes, hydrogels, and biodegradable scaffolds with microfluidic channels to reconstruct complex tissue interfaces and 3D microenvironments [43].

Porous polymer membranes (e.g., polycarbonate) act as selective permeability barriers in epithelial–endothelial coculture models. Their pore size and thickness determine the trans-membrane migration capacity of small molecules, proteins, and cells between different chambers, thereby directly affecting barrier function and transport efficiency. This plays a critical role in models of the BBB and gut–blood barrier [40].

Crosslinkable hydrogels are injected into specific chambers and gelled in situ to form 3D ECM scaffolds with tunable elastic modulus and pore structure. These scaffolds facilitate the maintenance of functional phenotypes of parenchymal organ cells (e.g., hepatocytes and cardiomyocytes), enabling OOC systems to possess in vivo-like 3D microenvironments based on the fluidic control capabilities of LOC systems [43].

Coating the surfaces of membranes and hydrogels with ECM proteins (e.g., collagen and fibronectin) improves the adhesion, spreading, and differentiation of specific cell types, enhancing the reconstruction of organ-specific structures and functions. This provides a reliable biomimetic foundation for subsequent introduction of RBCs and plasma components to study interactions after storage injury [43].

In OOC systems involving blood or RBC perfusion, the introduction of hydrophilic or anti-protein adsorption coatings on channel and membrane surfaces reduces the adhesion of plasma proteins and platelets, lowering the probability of microthrombosis and mechanical cell damage. This enables controllable regulation of blood–material interactions in organ systems extended from LOC systems [84].

##### Numerical simulation-aided design

Compared with LOCs that mainly perform single-use or short-term analytical tasks, OOCs typically require the maintenance of continuous perfusion and stable cellular functions over a time scale of days to weeks, which imposes higher requirements on structural durability and the anti-clogging capability of channels [43].

Under long-term perfusion conditions, multi-layer PDMS structures may undergo slight deformations due to stress relaxation or solvent permeation. Therefore, a balance between geometric parameters and material thickness must be struck during the device design phase to reconcile overall rigidity with the required elasticity, ensuring long-term stable biomimicry on the basis of the channel network inherited from LOCs [40].

For blood-related OOC models, channel cross-sectional dimensions and bending radii directly affect local shear stress distribution and the residence time of blood cells near interfaces. Surface burrs or geometric discontinuities generated during fabrication can act as triggers for mechanical damage to RBCs and platelet activation, rendering precise control over processing accuracy and surface quality particularly critical. This provides a reliable geometric prerequisite for the subsequent simulation of mechanical exposure of stored RBCs in the microcirculation [77].

Based on the microfabrication foundation shared by both LOC and OOC, numerical simulation has become a critical auxiliary approach for the structural design and process optimization of OOC. By computing the velocity field, shear stress, and solute concentration distribution under different channel layouts and flow rate conditions, design schemes closer to the target physiological range can be screened prior to fabrication, enabling quantitative bionic optimization of the channel framework inherited from LOC [96].

In OOC research, including lung, liver, and tumor models, computational fluid dynamics and multiphysics simulations are employed to determine the width, height, and layout of perfusion channels. This ensures shear stress levels and gradient distributions close to those in vivo, thereby providing a basis for maintaining cell functions and controlling drug exposure [96].

In models involving blood and RBCs, simulation-driven optimization of channel geometries helps reduce local extreme shear zones and stagnant regions. This minimizes the disruption of nonphysiological mechanical stress on cell morphology and function and provide a quantitative design tool for subsequent studies on the mechanical environment of stored RBCs during reperfusion and circulation [96].

Under long-term culture conditions, phenomena such as material leaching, surface aging, and biofilm formation may alter the on-chip microenvironment. The selection of low-leaching materials, optimization of surface treatment processes, and development of appropriate pre-rinsing and sterilization protocols can to a certain extent improve the stability and reproducibility of OOC in simulating complex blood–organ interactions, laying a platform foundation for the systematic characterization of RSL [43].

#### Fabrication of EOC

Building upon the aforementioned processes for OOC, including multilayer structure construction, multi-material integration, and 3D printing, EOC—as a typical specialized application of OOC in vascular biomimicry—requires process customization and personalized construction tailored to vascular physiological characteristics and hemodynamic requirements (Fig. 3C).

##### Prepatterned microchannel endothelialization

Using mature soft lithography processes for LOC/OOC, rectangular microchannels are molded on PDMS or glass substrates, coated with ECM, and then seeded with endothelial cells. This enables the rapid construction of perfusable vascular channels with a continuous endothelial lining, which is suitable for basic research such as shear stress response, barrier permeability, and drug delivery [114].

Such on-chip channels can provide stable laminar flow at low Reynolds numbers and precisely controllable solute gradients while being compatible with high-resolution imaging. They represent the most commonly used fabrication format in early EOC models [115].

In addition, high-resolution stereolithography and 2-photon lithography techniques enable direct engraving of complex 3D microscaffolds within photosensitive hydrogels. Following seeding and coating with endothelial cells, these techniques achieve high-precision reconstruction of organ-specific vascular geometries such as brain microvessels [116].

##### Sacrificial template-based cavity formation

The sacrifice template method is a core process for fabricating bionic circular cross-section channels in EOC. Metal needles, wires, or reversible gel inks are embedded as sacrificial templates into hydrogel matrices such as collagen and gelatin methacryloyl (GelMA). After cross-linking and solidification of the matrix, circular cross-section or branched microchannels are formed by extracting or dissolving the templates, followed by endothelialization of the cavity walls to complete the construction of EOC [73].

Collagen-based circular cross-section cavities prepared using needle-shaped templates can stably control diameters at 50 to 120 μm; after endothelialization, they maintain an intact endothelial layer and measurable barrier permeability under continuous perfusion for multiple days [117].

Sugar glass scaffolds fabricated via 3D printing and embedded in GelMA hydrogels can form multilevel branched perfusable networks inside the hydrogel after sugar glass dissolution. This approach establishes a unified bionic blood supply pathway for multi-organ-on-a-chip (MOC) systems and represents a key process for achieving hierarchical vascular networks [118].

Micromolding, 3D printing, and microfluidic spinning are 3 representative techniques for preparing perfusable channels in hydrogels, which can be flexibly selected according to requirements for channel scale, geometric complexity, material compatibility, and cell adaptability [119].

### Cell sources and configuration

The biomimetic functions and application value of OOC systems are ultimately realized through appropriate cell systems and rational cell configuration strategies. This constitutes the core step in transitioning OOC platforms from engineering design to biological function implementation.

#### Cell sources for OOC

##### Immortalized cell lines

Immortalized epithelial or endothelial cell lines are widely used to construct monolayer cell barriers in barrier-type OOC systems. Their stable availability, straightforward expansion, and lenient culture conditions make them particularly suitable for early process optimization and high-throughput preliminary drug screening [86].

In practical applications, these cell lines present distinct advantages in cost control and operational convenience, yet they deviate from primary cells in terms of gene expression profiles and functional maturity. Both immortalized cell lines and primary cells require precise matching with the on-chip microenvironmental conditions, and the functional discrepancies between them can be partially compensated for through approaches such as coculture or microenvironmental regulation [109].

##### Primary cells

Primary human-derived cells better recapitulate the in vivo physiological state in physiological relevance, transporter expression, and drug-metabolizing enzyme profiles, making them widely used in OOC construction demanding high predictive performance [109,120].

Nevertheless, primary cells suffer from pronounced batch-to-batch variability and limited proliferative capacity, and they are highly sensitive to microenvironmental conditions (e.g., shear stress, substrate stiffness, and ECM composition). This necessitates precise matching of their on-chip applications with the functional requirements and structural design of specific organs [121].

##### Adult stem cells and iPSCs

Human iPSCs and other adult stem cell sources enable the construction of patient-specific disease models and drug response platforms in OOCs, allowing the establishment of multiple tissue modules with distinct genetic backgrounds under the same microfluidic architecture [93]. These cells exhibit remarkable advantages in source accessibility and genetic editability, but they also face challenges including complex differentiation protocols, insufficient functional maturity, and poor batch-to-batch stability [109].

Furthermore, when culturing iPSC-derived organoids and 3D tissue constructs on OOCs, dedicated optimization is required for chamber dimensions, oxygen and nutrient transport pathways, and imaging channels to prevent central necrosis of tissues and achieve long-term online monitoring [121,122].

#### Cell selection for EOC

EOC represents a specialized vascular-biomimetic subtype of OOCs. Cell type selection and configuration directly govern their structural biomimetic fidelity and functional authenticity. This necessitates rational, goal-oriented cell selection tailored to specific research objectives and application scenarios.

##### Endothelial cell selection

Human umbilical vein endothelial cells (HUVECs) are extensively used for endothelialization of macro/meso-scale vascular channels due to their easy acquisition and strong expansion ability. However, they differ substantially from organ-specific microvascular endothelia (e.g., those of the brain, liver, and kidney) in transporters and tight junction protein profiles [123].

Human brain microvascular endothelial cells can develop a barrier phenotype characterized by high electrical resistance and low permeability when cultured under suitable shear stress within a 3D matrix. For this reason, these cells serve as the primary cell source for BBB models built on EOC platforms [116].

Pluripotent stem cell-derived endothelial cells can acquire phenotypes close to specific organ vascular beds through directed differentiation and microenvironmental regulation, serving as promising cell resources for constructing patient-specific and organ-specific EOC [124].

##### Blood/immune component configuration

Whole blood, platelet-rich plasma, or reconstituted cell suspensions can be introduced into the perfusion system to investigate the interactions between blood/immune components and vascular endothelium under quantified shear conditions. This is applicable to simulating vascular physiological and pathological processes such as thrombosis, leukocyte adhesion, and complement-related reactions [79].

## Application of Microfluidic Chips in RSL Studies

During cold storage, the morphology of RBCs undergoes substantial alterations; however, their deformability and relaxation capacity may not exhibit a marked decline within the routine blood bank storage period under conditions approximating capillary dimensions and physiological blood flow. This indicates that morphological observation alone is insufficient to fully reflect the functional RSL, and more sophisticated functional detection methods are required [6]. In transfusion medicine practice, the reduction in RBC deformability during storage impairs the microcirculatory passage ability of transfused RBCs and may exacerbate transfusion-related complications, thereby rendering quantitative assessment of the mechanical property changes of RBCs during storage of great importance [125].

Currently, the storage and transfusion of RBCs predominantly adopt the “first-in-first-out” principle, i.e., blood products are selected solely based on storage duration. Nevertheless, multi-omics studies have confirmed that the storage quality of RBCs presents remarkable heterogeneity, which is manifested in multiple aspects including donor individual differences, storage time, and processing conditions. This has also driven the transformation of transfusion medicine toward a precision transfusion model based on functional indicators and novel detection technologies [126].

Existing studies have formed a systematic understanding of the quality assessment methods for RBC products. Traditional assessment indicators cannot comprehensively cover the multidimensional storage lesion characteristics of RBCs in terms of mechanical properties, rheological properties, and microcirculatory function. In contrast, emerging microfluidic and chip technologies are regarded as potential supplementary tools to remedy the deficiencies of traditional indicators and improve the quality assessment system [127].

### Applications of LOC in RSL

#### Single-cell biophysical characterization

**Fig. 4.**
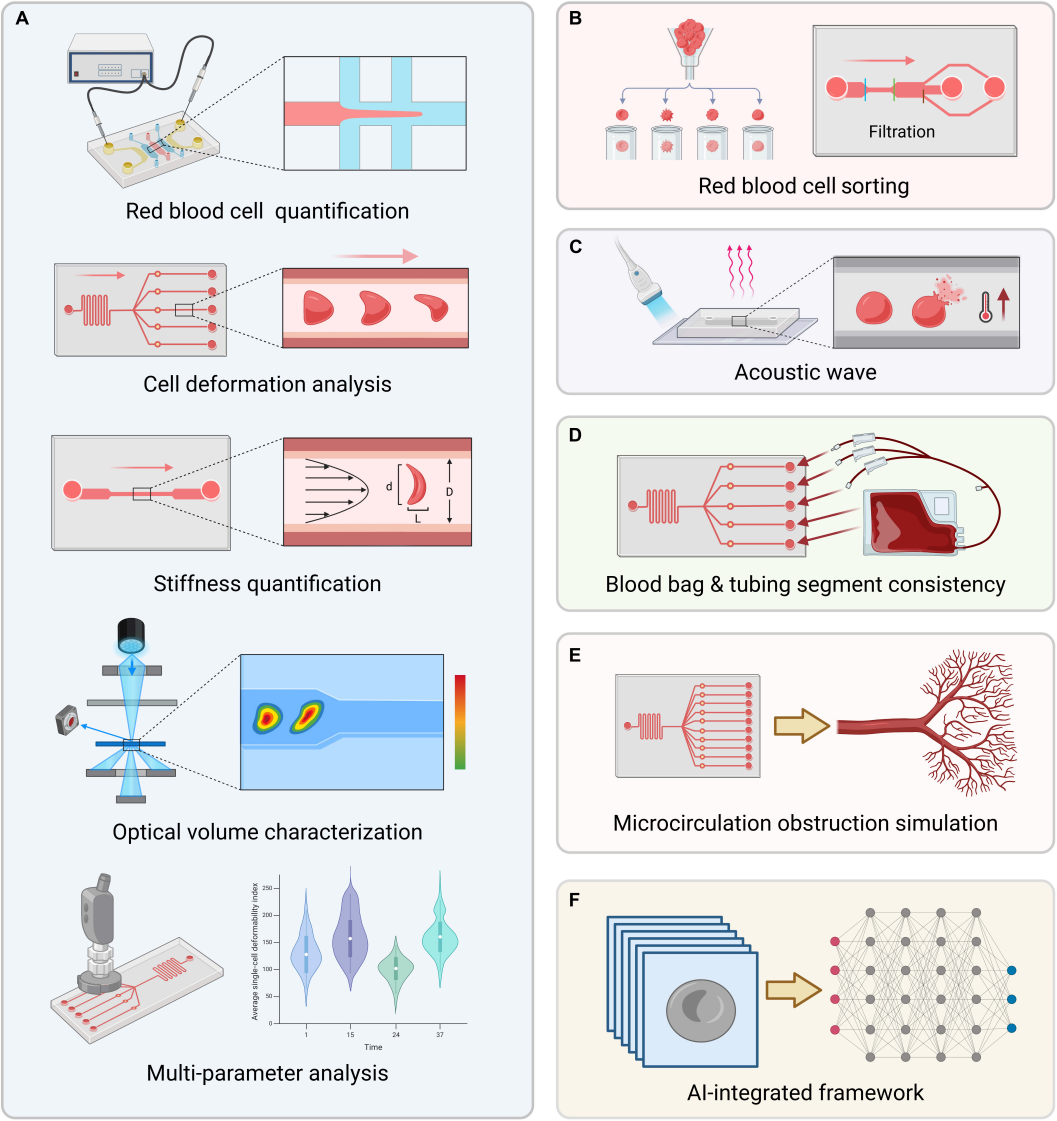
Multifunctional applications of LOC in RSL assessment. (A) Single-cell biophysical characterization including RBC quantification, deformation analysis, stiffness measurement, optical volume detection, and multi-parameter profiling. (B) RBC filtration, deformability-based sorting, and separation modules. (C) Hemolysis and membrane fragility evaluation based on SAW technology. (D) Stiffness quantification and consistency validation between blood bags and tubing segments. (E) Biomimetic simulation of microcirculatory obstruction and microvascular perfusion. (F) Integrated intelligent quality control system combining multi-parameter analysis and AI. Created in BioRender. Lei, Y. (2026) https://BioRender.com/5c6hvuq.

LOC techniques enable high-throughput, single-cell, and label-free quantitative analysis of the biophysical properties of stored RBCs under near-physiological conditions. These methods overcome the drawbacks of traditional bulk assays and support comprehensive, accurate characterization of RSL from multiple biophysical dimensions (Fig. 4A).

##### RBC quantification by impedance microfluidics

Microfluidic impedance chips based on polyelectrolytic gel electrodes (PGEs) enable rapid and precise quantification of RBCs, providing basic quantitative indicators for storage lesion research [128]. This technology adopts low-voltage direct-current (DC) impedance as the detection principle, capturing impedance signals when RBCs pass through PGEs on microchannel walls; the number of impedance peaks corresponds to RBC count, and peak amplitude reflects cell size. Detection requires only 2 μl of whole blood diluted 800-fold without pretreatments such as centrifugation or filtration. PGEs isolate metal electrodes from RBCs, avoiding cell damage and electrode fouling caused by high electric field gradients, while hydrodynamic focusing reduces cell coincidence effects. The counting results show an error of ≤10% compared with commercial clinical hematology analyzers, and detection is unaffected by cell flow rate or position in the channel, meeting the miniaturization and point-of-care rapid testing requirements of LOC systems [128].

##### Single-cell deformability and morphology

LOCs integrated with capillary-scale microchannels allow high-throughput characterization of deformation and relaxation behaviors of individual RBCs under near-physiological conditions. Morphological information can be captured simultaneously, thereby resolving the rheological properties of RBCs during blood bank storage at the single-cell level [6]. Continuous time-point detection of RBCs stored under standard blood bank conditions using such devices revealed no substantial changes in RBC deformability and relaxation kinetics in microchannels throughout the storage period, despite obvious morphological deterioration, indicating that morphological alterations alone do not necessarily lead to functional stiffening of RBCs in the microvascular environment [6].

Mechanical analysis of cold-stored RBCs using LOCs with similar capillary constriction structures demonstrated that the flow behavior of RBCs in microchannels can quantitatively characterize storage-related decreases in deformability, establishing a correlation between the concept of “RSL” and microfluidic mechanical phenotypes [129]. Further studies combining microfluidic technology with mathematical models deduced key mechanical parameters such as elasticity and viscoelasticity of RBCs by analyzing their confined flow behavior in microchannels, confirming that elasticity markedly increases, while deformability and normalized curvature decrease with prolonged storage, providing a parametric mechanical framework for quantifying RSL [130].

A portable LOC detection device named Erysense represents a typical application of capillary-like microchannel LOC. It employs 8 μm × 11 μm rectangular cross-section capillary-mimicking microchannels, combined with high-precision pressure control to achieve physiological flow rates of 0.1 to 10 mm/s. This effectively simulates the in vivo microcirculatory environment, accurately tracks the flow trajectory and morphological dynamic changes of single RBCs, and requires only sub-microliter blood samples, greatly reducing sample demand [131]. Integrated with high-speed imaging and custom image analysis scripts, this device accurately locates the centroid of RBCs and calculates flow velocity, thereby constructing an RBC morphological phase diagram. Healthy RBCs exhibit centrally flowing croissant shapes at low flow rates and transform into eccentrically flowing slipper shapes at increased flow rates. RBCs with storage lesions cannot form stable slipper shapes at a high flow rate of 10 mm/s due to decreased deformability, instead presenting more abnormally centrally flowing morphologies [131]. Researchers performed 10-week dynamic microfluidic detection on RBC concentrates refrigerated at 4 °C using this device, finding that the proportion of eccentric slipper-shaped RBCs at a high flow rate of 10 mm/s decreased substantially with prolonged storage, while the proportion of abnormally centrally flowing morphologies gradually increased, with obvious interdonor individual differences [131]. This result further confirms that capillary-like microchannels can effectively quantitatively characterize storage-related decreases in RBC deformability and precisely capture the interdonor heterogeneity of storage lesions, providing a more accurate technical approach for single-cell analysis of RSL.

A LOC-based RBC deformability detection system models peripheral vessel constriction and supports the study of RBC functions under pathological conditions [132]. This chip establishes the RBC deformability index (RDI) by measuring the normalized transit velocity and deformation degree of RBCs, and both intra-assay and inter-assay detection consistencies of this index are verified to be at a high level. Research on iron deficiency anemia found that RBC deformability in affected patients shows a marked increasing trend, and RDI is strongly negatively correlated with ferritin levels. The thinning, elliptical morphological changes, and reduced internal viscosity of RBCs induced by iron deficiency are the core causes of the abnormally increased deformability [132]. This study is the first to clarify the correlation between iron deficiency severity and RBC deformability, further expanding the application scenarios and clinical value of microfluidic chips with vessel-mimicking channels in the quantitative evaluation of RBC functions in hematological diseases.

##### RBC effective stiffness assessment

In a LOC device that mimics the folded transit of RBCs through capillaries, the introduction of a flow velocity-independent effective stiffness parameter enables a more robust quantitative assessment of the overall mechanical degradation of RBCs under physiological deformation modes [125]. Using this folding-mode chip, fresh RBCs and RBCs stored in blood banks for up to 6 weeks were tested separately. The results showed that the effective stiffness of RBCs increased substantially after approximately 1 week of storage as compared to fresh samples; moreover, the rate of stiffness rise accelerated further during 3 to 6 weeks of storage, indicating that mechanical damage can be detected in RBCs at the early stage of storage and accumulates substantially with storage time [125]. This study simultaneously measured the levels of S-nitrosothiols and ATP, and found that the time points of elevated effective stiffness were highly consistent with the decreasing trends of the above 2 classic biochemical indicators of storage. This establishes an experimental link between storage-related mechanical degradation of RBCs and impairments in energy metabolism and NO-associated signaling [125].

##### Optical volume characterization

In a microfluidic constriction channel integrated with quantitative phase microscopy and a high-refractive-index medium, real-time quantitative detection of optical volume changes can be performed during RBC transit through narrow channels, thereby indirectly reflecting the dynamic alterations in intracellular water content and internal density [133].

Analysis of RBCs from 2 subjects at different cold storage durations using this platform revealed that the magnitude of optical volume changes in RBCs under mechanical stress gradually decreased with prolonged cold storage, reaching a statistically significant difference by day 29 of storage, suggesting that storage injury impairs the transmembranous water exchange capacity and internal density regulation ability of RBCs under mechanical stress [133]. Based on these findings, the study proposed that the technical platform combining microfluidic mechanical stress and quantitative phase imaging can serve as a potential biophysical detection tool for investigating water homeostasis and membrane permeability changes during RBC cold storage, expanding the biophysical research dimension for characterizing RSL [133].

##### Multi-parameter biophysical analysis

An LOC system based on an artificial microvascular network, coupled with an intelligent automated particle tracking algorithm, enables high-throughput analysis of the entire RBC population at single-cell resolution. It accurately quantifies a single-cell deformability index derived from cell movement velocity while simultaneously obtaining effective size information for RBCs. Furthermore, it constructs a 2D biophysical map of RBC deformability versus size, analogous to flow cytometry density plots, through density-colored scatter analysis, thereby characterizing multi-parameter biophysical properties of RBCs [134].

This technical system has been successfully applied to RBC detection in healthy donors, patients with sickle cell disease (SCD), and patients with severe β-thalassemia major, as well as analysis of packed RBCs after 4 weeks of cold storage. It achieved high-sensitivity, accurate single-cell deformability measurements in all the above samples. Moreover, studies on stored RBCs confirmed that cold storage substantially reshapes the subpopulation distribution of RBCs in 2D deformability–size analysis, revealing a marked increase in functional heterogeneity of the RBC population induced by storage injury [134].

#### Deformability-based RBC sorting

Inherent interdonor variability exists in RBC deformability. Previous microfluidic studies using funnel-shaped microconstriction channels have confirmed that RBCs from different donors exhibit marked differences in susceptibility to oxidative damage, laying a solid theoretical foundation for subsequent RBC deformability sorting, trajectory tracking, and storage aging evaluation [135]. Supported by this theory, the LOC ratchet microfluidic device achieves precise single-cell RBC deformability sorting through a multi-stage microconstriction array [136]. By distinguishing the resistance differences of RBCs traversing microconstriction structures, the device diverts RBC populations from a single sample into different outlets to form distinct cell distribution profiles. Based on these profiles, a rigidity scoring system is established to enable quantitative characterization of RBC population deformability.

Using the aforementioned LOC sorting platform, researchers conducted the first longitudinal detection of RBCs cold-stored in standard test tubes to further explore interindividual differences in deformability. The results demonstrated that the “aging curves” of RBC deformability over cold storage time differed markedly across donors, whereas the aging curves of RBCs from the same donor across multiple donations were highly reproducible. This confirms that the ability of RBCs to resist cold storage lesions exhibits clear interindividual specificity [136]. The study also found that RBCs from some donors maintained deformability near the initial baseline after 2 weeks of cold storage, indicating the existence of “elite donors” in clinical practice who can provide long-storage, high-quality RBCs for sensitive transfusion recipients, offering a new strategy for precise blood matching [136].

To align with real clinical blood storage scenarios, the research team further extended the storage model from test tubes to blood bags. Using the same LOC ratchet device, deformability of RBC concentrates was evaluated every 14 d over a 56-d cold storage period. The results showed that within the current statutory 42-d storage limit, the rigidity scores of RBCs in most blood bags remained stable overall with no substantial deterioration in deformability. During the extended storage phase of 42 to 56 d, the rigidity deterioration rate of RBCs from different donors displayed marked interindividual differences, further verifying that inherent interindividual traits in deformability influence storage stability [137].

Building on these findings and targeting the clinical need for “purifying” stored blood prior to transfusion, related LOC research integrates microstructural filtration and hydrodynamic sorting technologies to selectively remove stiff and fragile RBC subpopulations that accumulate during blood storage. This technique reshapes the mechanical composition of RBC populations in vitro and effectively enhances the potential ability of RBCs to traverse the microcirculation [138]. Studies confirmed that the sorting technology exhibits superior purification efficacy for aged RBCs stored beyond 28 d. Compared with conventional blood washing methods, this technique not only minimizes mechanical damage to RBCs but also selectively eliminates deteriorated RBCs rather than merely removing plasma components, further highlighting its core application value in optimizing stored blood quality and improving transfusion safety and efficacy [138] (Fig. 4B).

#### Hemolysis and membrane fragility assessment

The surface acoustic wave (SAW)-based hemolysis detection platform applies combined acoustic and thermal stress within LOC channels. This defines the SAW-induced hemolysis temperature as a quantitative physical indicator of membrane fragility and hemolysis susceptibility in stored RBCs [139]. This method was used to test human RBCs stored for up to 42 d under cold conditions. The results showed that the SAW-induced hemolysis temperature exhibited reproducible changes over storage time and distinct trajectories across different donors, reflecting the association between donor-specific membrane mechanical robustness and metabolic profiles [139].

Integrated metabolomics analysis in the study revealed specific coupling patterns between SAW-induced hemolysis characteristics and RBC metabolic status. This confirms that the platform can serve as a candidate tool for on-site rapid assessment of stored RBC quality prior to transfusion, effectively bridging the gap between traditional metabolic markers and functional RBC membrane fragility detection [139] (Fig. 4C).

#### Clinical consistency validation

Rigidity scores of RBC concentrates in blood bags and their corresponding tubing segments were determined at various time points during up to 8 weeks of cold storage using a LOC “ratchet” device [140]. It demonstrated that deformability indices of segment samples were highly consistent with those of RBCs in blood bags at all time points.

Conventional hematological parameters, including mean corpuscular volume (MCV), red cell distribution width (RDW), mean cell hemoglobin (MCH), and mean corpuscular hemoglobin concentration (MCHC), were measured concurrently in the same study. The results revealed strong correlations of these parameters between blood bag and segment samples, whereas hemolysis showed poor correlation between the 2 sample types. This indicates that tubing segments serve as suitable nondestructive representative samples for deformability- and volume-related parameters, but are inappropriate for evaluating hemolysis indicators [140].

To enhance the reproducibility of microfluidic deformability measurements, a capillary-mimicking LOC study introduced a standardized flow standard to calibrate the flow resistance and operating parameters of the device, thereby markedly improving the consistency of stored RBC deformability assessments across different experimental batches and chips [141] (Fig. 4D).

#### Microcirculation and occlusion simulation

Capillary network-mimicking microfluidic chips represent the early research foundation for microcirculatory function simulation. Previous studies integrated hydrodynamics and circuit analysis to establish a motion model of RBCs in capillary networks, analogizing fluid pressure drop, flow resistance, and flow rate to electric potential, electrical resistance, and electric current, respectively, enabling quantitative calculation of flow field changes during microvascular occlusion by RBCs [142]. Based on this model, the study fabricated a PDMS-based capillary network-mimicking chip via soft lithography. By simulating in vivo capillary constraints through biomimetic channel structures and regulating flow rates, the dynamic processes of RBC occlusion–deformation–perfusion were visualized [142]. Experiments confirmed that this chip could distinguish healthy RBCs from those with impaired deformability via changes in pressure drop, and required only trace blood samples for detection, laying the core foundation of network modeling and chip fabrication for the subsequent design and development of chips simulating microcirculatory occlusion [142].

A biomimetic network LOC constructed based on the “sheet flow” characteristics of pulmonary alveolar capillaries further enabled accurate characterization of RBC dynamics at physiological hematocrit. This chip employed staggered array micropillars to simulate the dense mesh structure of pulmonary capillaries, restoring the physiological capillary scale of 3 to 10 μm. Using RBC tracking velocimetry, 3 heterogeneous migration patterns of RBCs in pulmonary capillary networks were identified: quasi-straight flow, local trapping, and lateral translocation. Additionally, capillary segment size was identified as the key determinant of network hydrodynamic resistance. The chip also verified that despite marked heterogeneity in RBC dynamics at the microscale, the macroscopic flow rate–pressure drop relationship of the network remained linear, providing critical experimental evidence for hydrodynamic modeling specific to pulmonary microcirculation [143].

In an LOC channel network composed of multi-scale micropillar arrays, a gradient microcapillary structure was constructed in a single path by gradually reducing the pore size from 20 μm to 4 μm, and arteriovenous anastomotic shunts were simulated via bypass channels. This allowed quantification of RBC occlusion in “capillaries” of different sizes without blocking the overall pathway [144]. When analyzing abnormally deformable RBCs, this “OcclusionChip” demonstrated that stiff cells were preferentially retained at larger upstream pores, while relatively soft pathological cells occluded at smaller pores, forming a size-dependent “microvascular occlusion index” that can serve as a functional characterization of RBC-mediated microcirculatory perfusion impairment [144].

A high-throughput automatic tracking system based on a microvascular network model can also convert single-cell-level deformability information into flow field distributions and transit time spectra in a simulated microcirculatory environment by measuring the motion trajectories and velocities of RBCs in complex microchannel structures, thereby resolving the subpopulation characteristics of impaired microcirculatory function in stored RBCs [134] (Fig. 4E).

#### AI-integrated microfluidic quality control

Microfluidic RBC deformability detection and imaging analysis technologies represent emerging quality control tools for stored blood in the pretransfusion stage. The integration of AI into this detection system complements traditional biochemical indicators and multi-omics detection data in dimensionality, yielding unique technical value and practical potential in combined applications [7,145,146]. Leveraging AI algorithms such as deep learning and unsupervised clustering enables intelligent end-to-end processing of high-dimensional RBC data generated by microfluidic imaging—from morphological recognition to functional feature parsing—markedly improving the efficiency, accuracy, and standardization of detection and analysis while effectively compensating for the limitations of manual analysis [147].

Label-free imaging based on imaging flow cytometry combined with deep learning allows automated classification of 6 morphological states associated with storage lesions in RBCs across a 42-d storage period without additional staining. Analytical models constructed by extracting deep visual features of RBCs can capture the continuous evolutionary pattern of their morphology with storage duration, providing core technical support and data evidence for inferring the remaining effective storage time of stored blood [148].

Using RBC deformability sorted by the LOC “ratchet” device as the ground-truth label, training a convolutional neural network on single-cell microscopic images achieves approximately 81% accuracy in classifying single-cell deformability. At the sample level, the prediction error of the overall deformability score can be controlled within approximately 10% [149].

The open-source AI analysis software iCLOTS provides a mature tool for intelligent analysis of microfluidic RBC detection. Integrating computer vision, machine learning, and data science algorithms, it automates processing of microfluidic microscopic imaging data of RBCs, enabling single-cell tracking, adhesion analysis, velocity profile construction, and multiscale microfluidic aggregation analysis. The embedded *k*-means unsupervised clustering algorithm can naturally group high-dimensional detection data, accurately identify RBC subpopulations, distinguish healthy RBCs from those with storage lesions, and quantify functional heterogeneity caused by storage lesions [150]. As a programming-free, standalone open-source system, the software directly imports microfluidic image and video data, completes analysis via interactive parameter adjustment, and outputs quantitative values and visual charts. It integrates multidimensional indicators including RBC morphology, flow velocity, and deformability [150], providing a standardized, high-throughput AI analysis solution for RSL assessment, and effectively reducing errors and biases in manual analysis.

For clinical applications, a multi-parameter functional evaluation platform centered on LOC should incorporate donor biological characteristics and omics markers to support the transformation of transfusion strategies from “usage by storage time” to “allocation by functional quality”, thereby exerting decision-supporting roles in actual transfusion workflows [126] (Fig. 4F).

### Applications of OOC in RSL

Based on LOC technology, OOC upgrades the conventional planar fluidic system of LOC into a vascular-mimetic functional device with reconfigurable blood–endothelium interface (i.e., EOC) by constructing a continuous, perfusable endothelial luminal surface inside microchannels. This enables in-depth dissection of bidirectional interactions between blood and endothelial cells at scales where RBC size is comparable to vessel diameter and blood flow exhibits typical microcirculatory characteristics [73]. EOC closely recapitulates the in vivo microcirculatory environment in terms of geometric dimensions (vessel diameter < 100 μm), shear stress range, and cellular composition, making it suitable for investigating pathophysiological processes closely associated with RBC function, including RBC–endothelium interactions, microvascular resistance regulation, and microthrombus formation [73].

In such devices, microchannels with regular or complex geometries are fabricated in rigid substrates or hydrogels and fully endothelialized on the luminal surface. This maintains precise control over flow field and shear stress while introducing authentic cellular interfaces and endothelial surface layers, rendering RBC-related experiments more physiologically relevant to the in vivo vascular milieu [151]. Compared with rigid microchannels lacking endothelium, the endothelium and its surface layer in EOC markedly alter RBC near-wall distribution, hydraulic resistance, and microrheological parameters of aggregation/disaggregation. It also reconstructs complex phenomena such as RBC adhesion, microvascular occlusion, and hemolytic injury in disease models, providing a methodological foundation for extending functional assessment of RBC storage injury to a near-physiological vascular environment [152].

A variety of EOCs have been developed, and each structural design exhibits unique advantages alongside intrinsic technical limitations. To systematically summarize and quantitatively analyze the core architectures, quantifiable readouts, advantages, and limitations of representative published EOC platforms, a comparative overview is presented in Table 1.

**Table 1.** Summary of EOC platforms for RSL and related research

Platform type	Structural design	Cell source	Biomimetic parameters	RSL and functional readouts	Advantages	Limitations	References
Clinical RSL high-throughput assay chips	Rectangular parallel channels, fibronectin coating, 4-h rapid endothelialization	HUVEC	1 dyn/cm^2^ shear; heme/hypoxia/thrombin stimulation; short perfusion	RBC adhesion rate, CSS, endothelial adhesion molecule expression	Standardized clinical detection; direct hypoxic storage RBC adhesion quantification; simple workflow	Single-dimensional RBC index only; no long-term culture; rectangular nonphysiological lumen	[153–155,158]
Pathological geometry modelling chips	Custom tunable microchannels, stenosis/bifurcation geometry	HUVEC, HAEC, HMVEC, HLMVEC	Shear 0–220 dyn/cm^2^; adjustable osmolarity/hypoxia; laminar flow	RBC transit time, microchannel occlusion, VWF morphology, platelet adhesion	Arbitrary pathological vessel geometry; whole blood and drug screening; low-cost PDMS fabrication	Static culture cannot mimic long-term endothelial remodeling; rectangular channel flow bias	[157,159,162,188]
3D hydrogel-based long-culture chips	Hydrogel 3D lumens, multi-cell coculture space	HUVEC, HLMVEC, hiPSC-EC, pericytes, THP macrophages	Shear 0.1–10 dyn/cm^2^; sustained perfusion up to 30 d	Endothelial permeability, RBC fragmentation, thrombus, angiogenesis sprouting	Month-long chronic injury simulation; ECM remodeling; immune-endothelial crosstalk	Hydrogel weak mechanical strength; minimum channel 20 μm; matrix degradation risk	[152,161,165]
Patient-derived autologous endothelial chips	Conventional microchannels, fibronectin for primary cell adhesion, whole blood perfusion	Human/porcine BOEC, patient PAEC, HUVEC control	Tunable arterial/venous shear; TNF-α/histamine stimulation; max 18-h culture	Platelet/fibrin deposition, endothelial gap leakage	Autologous blood-derived EC, no biopsy; personalized inflammation modeling	Short culture window; no dedicated RSL testing; rectangular lumen mismatch	[163,189]
Hierarchical microcirculation chips	Self-assembled artery–capillary–vein integrated network	HUVEC, lung fibroblast, smooth muscle	Dual pressure gradient (capillary bed 200 Pa, arteriole channel 60 Pa)	Vascular permeability, cell extravasation, vasoconstriction	Unique complete microcirculation architecture; multi-scale cell behavior analysis	Self-assembled capillary heterogeneity; low arterial flow; no RSL research	[166]

EOC, endothelium-on-a-chip; RSL, red blood cell storage lesion; RBC, red blood cell; CSS, critical shear stress; VWF, von Willebrand factor; HUVEC, human umbilical vein endothelial cells; HLMVEC, human lung microvascular endothelial cells; HAEC, human aortic endothelial cells; HMVEC, human microvascular endothelial cells; hiPSC-EC, human induced pluripotent stem cell-derived endothelial cells; HBVPCs, human brain vascular pericytes; BOECs, blood-derived endothelial outgrowth cells; PAEC, human pulmonary artery endothelial cells; THP-1, human acute monocytic leukemia cell line; NHLFs, human lung fibroblasts; SMCs, human lung smooth muscle cells; ECM, extracellular matrix; TNF-α, tumor necrosis factor-α

#### EOC for RBC rheological evaluation

**Fig. 5.**
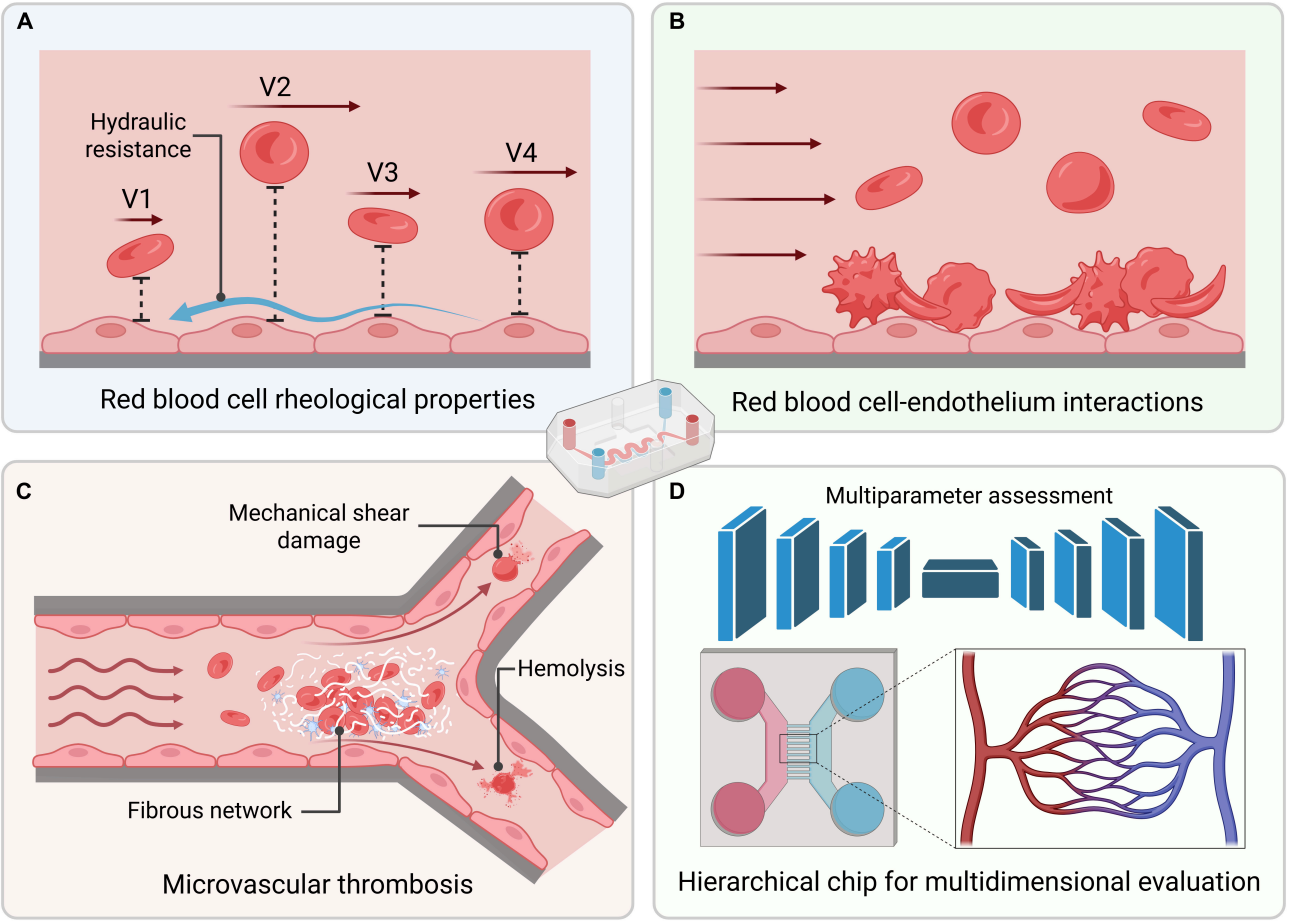
Applications of OOC (EOC) in evaluating RSL. (A) RBC rheological property detection. (B) RBC–endothelium interaction analysis. (C) Modeling of microvascular thrombosis, mechanical shear injury, and hemolysis. (D) Multiparameter comprehensive evaluation of RSL. Created in BioRender. Lei, Y. (2026) https://BioRender.com/vljqb7u.

EOC platforms reconstruct the physiological endothelial interface and luminal microenvironment, enabling quantitative analysis of RBC rheological behaviors under biomimetic shear conditions. These systems clarify how the endothelial layer regulates flow characteristics, RBC aggregation, and microrheological parameters, providing a physiologically relevant platform for evaluating RBC functional impairments caused by storage lesions (Fig. 5A).

##### Endothelial surface layer and flow regulation

Confluent endothelial monolayers are established within regular rectangular microchannels and characterized by confocal imaging. A continuous endothelial surface layer extending over the entire luminal surface was clearly visualized, with a thickness of approximately 600 nm—consistent with the thickness of the glycocalyx in in vivo microcapillaries. This provides a solid structural foundation for in-depth investigations into RBC–glycocalyx interactions within chip-based systems [151].

In the same endothelialized microchannels, measurements of pressure drop–flow relationships and RBC velocity fields revealed that the presence of endothelium substantially increased hydraulic resistance compared to non-endothelialized channels. Additionally, the velocity distribution and motion trajectories of RBCs in the near-wall region were markedly altered, indicating that the endothelium and its surface layer reshape near-wall flow characteristics and apparent viscosity of RBCs at the micrometer scale [151]. Within EOC systems, these parameters of hydraulic resistance and near-wall RBC velocity fields are closely correlated with viscosity and microcirculatory flow capacity—key indicators targeted in conventional studies of RBC storage injury. This establishes a bridge linking the readouts of EOC assays to the core features of storage injury [151].

##### RBC aggregation and disaggregation

An EOC was constructed based on a slit-shaped microchannel with HUVECs cultured on its luminal surface. Combined with laser aggregometry, the changes in backscattered light intensity of whole blood containing RBCs under different shear stresses were recorded. The hydrodynamic strength and disaggregation behavior of RBC aggregates can be quantitatively evaluated using a single parameter—the critical shear stress [153]. A comparison of experimental results between bare channels and endothelium-covered channels reveals that the presence of endothelial cells leads to a marked decrease in the critical shear stress of RBC aggregates, along with a marked improvement in measurement accuracy. This phenomenon indicates that the authentic endothelial interface and its microscopic morphology alter the local shear field and the RBC–wall lubrication state, thereby affecting the microrheological detection results of RBC aggregation based on microfluidic technology [153].

This critical shear stress index directly reflects the alteration in aggregation tendency during RSL, providing a novel detection dimension for quantifying RBC dysfunction induced by storage lesion [153]. In single-RBC-scale microfluidic studies, multifunctional chip platforms integrating microscopic imaging, tomographic analysis, spectroscopic analysis, trapping arrays, and aggregation/agglutination modules have been successfully developed. These platforms enable precise assessment of key parameters such as RBC deformability, aggregability, and electrical properties. Some of these platforms have been applied to detect changes in the electrical properties of stored RBCs, offering reliable technical support for quantifying the impacts of storage conditions and storage duration on the physical properties of RBCs [8].

#### RBC–endothelium interaction analysis

EOC models enable real-time, quantitative, and shear-controlled analysis of RBC adhesion to the vascular endothelium under physiological and pathological conditions. These platforms reveal how vascular geometry, endothelial activation, biophysical properties of RBCs, and external interventions regulate RBC–endothelium interactions, offering a physiologically relevant tool for evaluating adhesion abnormalities caused by RSL (Fig. 5B).

##### RBC adhesion under physiological and pathological conditions

The EOC based on linear microchannels enables the perfusion of RBCs under physiological shear conditions. By quantitatively counting the number of adherent cells per unit area, a functional evaluation system applicable to the microvascular scale is established, which can characterize RBC adhesion phenotypes and individual differences [154]. When endothelial cells in the same chip platform are exposed to pathological concentrations of free heme, the adhesion of RBCs to the endothelium is markedly enhanced. Moreover, the adhesion intensity varies among patients and correlates with plasma hemolytic marker levels and clinical disease severity, demonstrating that this EOC can sensitively reflect functional differences in the hemolysis–endothelial activation–RBC adhesion pathway [154].

The standardized EOC platform allows for the quantitative analysis of the adhesion behavior of stored RBCs to human microvascular endothelial cells under controlled shear conditions. Studies have confirmed that different oxygen environments during storage alter the endothelial adhesion properties of RBCs, which are directly associated with the storage lesion phenotype [155]. This method provides an experimental basis for evaluating the adhesiveness of stored RBCs under physiologically relevant conditions and can also be used to screen RBC storage strategies that reduce the risk of post-transfusion endothelial activation and microvascular occlusion.

##### Vascular geometry-mediated adhesion regulation

Endothelial monolayers were established in complex vascular geometries (including stenoses, aneurysms, and bifurcations) fabricated with PDMS [156]. Combined with fluid dynamic analysis and immunofluorescence imaging, a correlation was established between the local wall shear stress distribution and the spatial expression of adhesion molecules such as vascular cell adhesion molecule-1. It was revealed that the shear environment dictated by vascular geometry can modulate the adhesion potential of blood cells including RBCs by regulating endothelial adhesion molecule expression [156]. In identical stenosis–bifurcation EOC, the presence of endothelial cells reduced platelet adhesion at stenotic sites while increasing RBC adhesion in bifurcation regions, indicating that the same endothelial layer exhibits cell-type and spatial-position specificity in regulating the adhesion behavior of different blood cells under distinct geometries and shear environments [156].

##### Biophysical property–adhesion correlation

Another category of EOC models for RBC research enables quantitative correlation of RBC biophysical properties and adhesive function within a single system by modulating extracellular fluid tonicity to alter RBC volume and deformability and measuring corresponding adhesive behaviors on endothelial surfaces under flow. This highlights the advantage of EOC in simultaneously resolving cellular biophysical properties and functional phenotypes [157]. This technical framework is also applicable to stored RBC research, allowing precise correlation of morphological and deformability changes during storage with adhesive and passage performance of RBCs in the microvascular environment [157].

##### Intervention effects on RBC–endothelium adhesion

Thrombin stimulation assays performed in EOC allow direct observation of thrombin-mediated endothelial contraction and injury, accompanied by quantitatively enhanced RBC adhesion to the damaged endothelium. Conversely, combined treatment with human antithrombin III markedly reduces RBC adhesion, validating that EOC can be used to evaluate the regulatory effects of anticoagulant and anti-inflammatory interventions on RBC–endothelium adhesion [158].

#### Microvascular thrombosis and occlusion modeling

EOC systems enable in vitro recapitulation of pathophysiological processes including microvascular occlusion, thrombosis, and mechanical damage under controlled hemodynamic conditions. These platforms clarify the roles of stored RBCs in thrombotic events and endothelial barrier dysfunction, providing a reliable experimental system for evaluating microcirculatory risks associated with RSL (Fig. 5C).

##### In vitro recapitulation of occlusion and thrombosis

By perfusing patient-derived RBCs or whole blood through microchannels with stenotic and bifurcated geometries in EOC, and monitoring channel patency and thrombotic progression in real time, disease-relevant microvascular occlusion and thrombosis can be recapitulated in vitro. This enables systematic dissection of the mechanisms by which RBCs contribute to microvascular obstruction under controlled conditions [159]. Hydrogel-based thromboinflammation EOC models inflammatory microenvironments to investigate thrombus formation and resolution, defining the regulatory roles of inflammatory factors in thrombus progression and endothelial injury. Studies in SCD using this platform confirm that neutrophil–platelet aggregates in patient blood directly trigger microvascular occlusion and impair endothelial barrier function [160].

3D EOC constructed from interpenetrating polymer network hydrogels support the formation of reversible focal flow obstructions by SCD RBCs or Plasmodium-infected RBCs in localized microchannels under flow conditions. Combined with transendothelial permeability assays using fluorescent tracers, delayed and reversible endothelial hyperpermeability induced by exogenous free heme can be visualized. This single platform directly presents the coupled relationships between pathological biophysical properties of RBCs, release of hemolytic products, and endothelial barrier dysfunction [152]. This approach simultaneously captures RBC-induced reversible microvascular occlusion and time-dependent endothelial permeability alterations elicited by free heme, offering a readily translatable experimental paradigm for introducing stored RBCs into engineered chip architectures and systematically assessing their microcirculatory function [152].

##### Thrombotic mechanisms and RBC mechanical injury

Engineered 3D endothelialized microvessels constructed on collagen scaffolds have enabled in vitro studies of intravascular thrombosis and associated microvascular processes under controlled flow fields and complex geometries, laying a foundational model for further delineating shear-dependent thrombotic mechanisms [161]. In EOC with curved and sharp-turn structures fabricated within 3D collagen matrices, von Willebrand factor (VWF) secreted by activated endothelial cells self-assembles into fibrous and web-like structures in regions of high shear stress or abrupt flow velocity changes, with enhanced assembly in microvessels ≤300 μm in diameter. This provides an in vitro platform with high spatial resolution for investigating macromolecule-mediated thrombosis at the microvascular scale [162].

Within these chips, the fibrous and web-like structures formed by VWF simultaneously capture platelets, leukocytes, and RBCs, triggering focal blood flow obstruction. Under certain conditions, they also inflict mechanical shear damage on passing RBCs, successfully recapitulating microvascular thrombosis and RBC mechanical disruption under high shear and geometric discontinuity in vitro [162]. Observing this process permits precise evaluation of RBC mechanical tolerance and hemolytic susceptibility in pathologically relevant environments characterized by high shear and geometric discontinuity. These measurements directly correlate with the functional consequences of increased mechanical fragility of RBCs following storage lesions, further refining the assessment system for storage lesions [162].

##### Diverse EOC models for thrombosis research

Perfusion of whole blood containing RBCs through linear microchannel EOC lined with primary human endothelial cells induces platelet-rich thrombus formation at physiologically relevant shear rates. Real-time imaging-based quantitative analysis of platelet adhesion area and fibrin deposition extent enables systematic assessment of thrombotic dynamics in the presence of endothelium, providing a reliable platform for antithrombotic drug screening involving RBC [163].

EOC constructed with chemically fixed human vascular endothelium that retains core hemostatic regulatory functions allow rapid detection of thrombosis and platelet function using small volumes of whole blood. This platform can be applied to analyze antiplatelet drug effects, demonstrating the clinical diagnostic feasibility of fixed endothelialized chips [164].

Perfusion of whole blood with RBCs through 3D EOC integrated with macrophages effectively induces intravascular coagulation and fibrin clot formation [165]. This model resolves the intercellular crosstalk among immune cells, endothelial cells, and blood components in the thrombo-inflammatory cascade on a single platform, providing a physiologically relevant in vitro system for exploring the synergistic mechanisms of thrombo-inflammation.

#### Hierarchical EOC for multidimensional assessment

The hierarchical EOC constructed by combining viscous finger patterning and self-assembly enables compartmentalized functional detection of different microvascular segments (arterioles, capillaries, and venules) [166]. This model can precisely measure the hydraulic resistance and transendothelial permeability of the entire microvasculature and each compartment, and quantitatively analyze the arrest and extravasation efficiency of circulating cells in vascular segments with distinct shear stress levels under tunable flow fields and shear stress conditions. Furthermore, it can synchronously record the regulatory effect of vascular smooth muscle cell-mediated vasoconstriction on microvascular flow fields and the microcirculatory behavior of circulating cells [166]. This technique extends the functional evaluation of RSL from a single vascular scale/single detection index to a multi-segment, multi-parameter comprehensive assessment within a hierarchical microvascular network. It can more comprehensively simulate the overall flow behavior of stored RBCs in the in vivo microcirculation, establishing a more systematic technical platform for correlating storage lesions with functional abnormalities of RBCs in different microvascular segments.

In EOC research centered on the blood–endothelium interface, reconstructing perfusable microvasculature with precisely defined geometric structures and shear conditions has fully demonstrated the core capability of such chips in dissecting bidirectional signal transduction between blood and endothelium under homeostasis. This lays a solid methodological foundation for the future comprehensive evaluation of the interactions between stored RBCs and endothelium, as well as the resulting microcirculatory functional changes, under near-physiological in vivo conditions [79] (Fig. 5D).

## Future Outlook

Current platforms possess prominent merits across core evaluation dimensions including physiological mimicry, multi-index detection, quantitative measurement, experimental throughput, and manufacturing reproducibility (Table 2). However, multiple interwoven unresolved technical and translational obstacles remain, including limited long-term coculture durability, incomplete biomimetic vascular topology, low detection sensitivity for trace injury biomarkers, insufficient experimental throughput, absence of unified quantitative grading thresholds, and nonstandardized chip fabrication workflows. Further technical iteration and systematic optimization are therefore required to fully unlock the translational potential of EOC and related microfluidic systems for RSL research and clinical transfusion practice, with multiple promising developmental directions elaborated below.

**Table 2.** Performance evaluation of EOC in RSL research

Evaluation dimension	Current status	Advantages	Limitations	References
Physiological fidelity	In vivo physiology recapitulation; core RSL pathologies reproduction; multi-factor process integration; vascular biomechanics mimicry	Higher physiological relevance; precise pathological recapitulation; multi-cell source compatibility	Short-term culture system; incomplete vascular structure	[152,154,155,159,166]
Detection dimensions	Multi-indicator simultaneous detection; RSL core indicator measurement; multi-level functional analysis; dynamic cell behavior visualization	Integrated multi-pathology detection; synchronized multi-function detection; real-time pathological visualization	Endpoint-based detection majority; low-abundance indicator insensitivity; trace factor detection challenge	[154,155,159,163,188]
Quantification capability	Core RSL indicator quantification; RSL difference quantification; intervention effect quantification	Higher quantification accuracy; clinical phenotype correlation; minimal sample volume requirement	Unified threshold absence	[154,155,159]
Throughput and clinical adaptability	Clinical sample compatibility; micro-sample detection support; clinical RSL scenario recapitulation	Direct clinical sample interface; pre-transfusion quality assessment; individualized susceptibility evaluation	Low throughput property; high technical requirement; long detection cycle	[154,155]
Technical standardization and reproducibility	Standardized protocol establishment; reproducible chip fabrication; good assay reproducibility; standardized assay formulation	Reduced experimental variability; superior reproducibility; guaranteed batch consistency	Insufficient fabrication standardization; unified standard absence	[154,155]

EOC, endothelium-on-a-chip; RSL, red blood cell storage lesion; RBC, red blood cell; SOP, standard operation procedure; QC, quality control

### Vascularized MOC for RSL research

As an advanced form of microfluidic biomimetic technology, vascularized MOC provide an innovative platform for simulating in vivo interorgan physiological and pathological interactions by constructing a multi-tissue/organ connection system with selectively permeable endothelial barriers [167–169]. Studies have successfully achieved long-term stable in vitro culture of mature tissues such as heart, liver, bone, and skin using MOC systems [170–172]. Its core advantages lie in connecting tissue-specific microenvironments through vascular channels and maintaining tissue phenotypic stability via endothelial barriers, enabling accurate recapitulation of in vivo pharmacokinetic/pharmacodynamic profiles of drugs and interorgan signal communication [170,173,174]. The capabilities of this system, including simulating trans-tissue substance exchange, regulating selective infiltration of immune cells, and maintaining long-term phenotypes, provide critical technical support for dissecting the systemic effects of RSL and are expected to break through the bottleneck of traditional single-organ models in simulating multi-organ interactive effects after RBC transfusion.

Current EOC used for RBC-related research are mostly constructed based on single-cell or single-tissue interfaces. Although they can simulate local microcirculatory mechanical environments and barrier functions, they have obvious limitations. Notably, they cannot recapitulate multi-organ cascade reactions induced by RSL, fail to simulate the synergistic effects of multiple systems such as liver metabolism, vascular endothelial response, and immune cell clearance, and lack the ability to integrate systemic physiological and pathological feedback after RBC transfusion [175]. Such single-cell chips can only characterize local mechanical or barrier interaction properties of storage-lesioned RBCs, and cannot comprehensively reveal critical clinical issues such as systemic oxidative stress, iron overload, and multi-organ functional impairment caused by these cells.

**Fig. 6.**
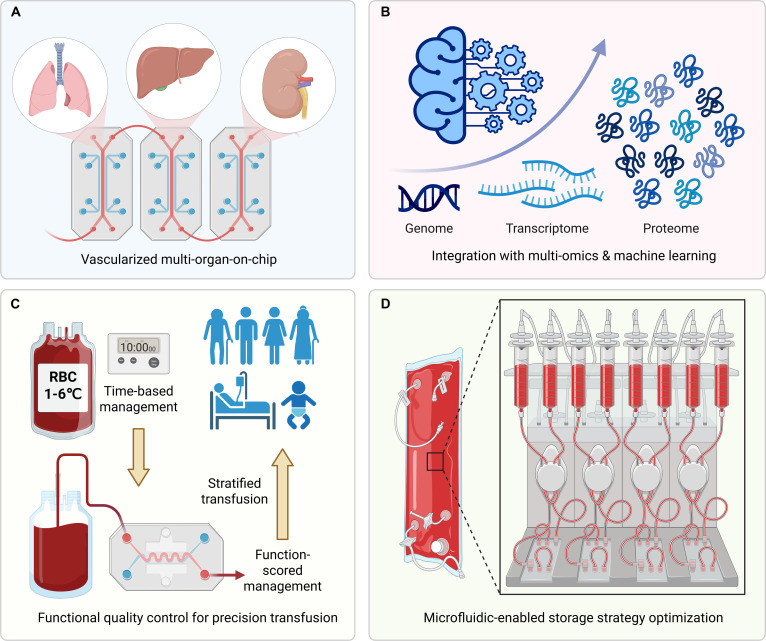
Future development directions of microfluidic technology in RSL quality control and precision transfusion. (A) Vascularized MOC for systemic pathophysiological simulation. (B) Integration with multi-omics and machine learning for intelligent quality prediction. (C) Shift from time-based to function-based management for stratified precision transfusion. (D) Microfluidic-optimized storage strategies and intelligent blood storage microreactors. Created in BioRender. Lei, Y. (2026) https://BioRender.com/6fmh65e.

In the future, vascularized MOC technology, and further advanced body-on-a-chip (BOC) systems, can be extrapolated to the field of RSL research. Leveraging the advantages of multi-tissue coculture of this technology, an integrated model containing key organs such as vascular endothelium, liver, kidney, and lung can be constructed to simulate the interactions between storage-lesioned RBCs and multiple organs after transfusion. Modular stacked MOC architectures with decoupled independent perfusion loops will be developed to extend continuous stable coculture cycles, alongside integrated hierarchical arteriole–capillary–venule channel networks that fully reconstruct the complete multi-scale vascular topology missing from conventional single-channel chips, resolving the short culture window and oversimplified vessel geometry that restrict comprehensive long-term pathological observation. Embedded dual-circuit substance enrichment modules will also be incorporated into the systemic circulation loop to boost the capture and measurable concentration of low-abundance circulating injury mediators, allowing sensitive tracking of subtle molecular damage signals generated during RBC storage. Leveraging its capabilities of endothelial barrier regulation and trans-organ signal communication, MOC platforms can accurately dissect the pathogenic mechanisms of injury products, such as free hemoglobin and RBC-derived microvesicles, and their distribution. By regulating the mechanical microenvironment and integrating multiple tissues in MOC, complex RSL-induced pathological processes, such as vasoconstriction, microthrombosis, and organ perfusion abnormalities, can be recapitulated in vitro. This technological expansion will advance RSL research from the local cellular level to systematic mechanistic dissection, providing a novel technical pathway for optimizing blood bank storage strategies and establishing a precise transfusion evaluation system (Fig. 6A).

### Integration with multi-omics and machine learning

The rapid advancement of multi-omics technologies has established systematic associations among donor genetic backgrounds, environmental exposure factors, and RBC storage quality. Through multidimensional analyses including genomics, metabolomics, proteomics, and lipidomics, key molecular signatures and regulatory pathways influencing RBC hemolysis rate and post-transfusion in vivo recovery have been identified [176]. Machine learning, a cross-learning method integrating computer science and statistics, boasts unique strengths in processing complex datasets [177,178]; so, leveraging its capacity to decipher high-dimensional omics data, this analytical framework accurately screens molecular patterns associated with RSL and transfusion clinical outcomes from massive metabolic and proteomic features. Meanwhile, algorithms such as random forest and lasso regression enable efficient identification of storage quality biomarkers. Furthermore, machine learning provides targeted design strategies for the development of novel blood storage additives via high-content screening of on-chip cell morphology and simulation models of RBC membrane mechanics [176], emerging as a core technological pillar driving transfusion medicine from empirical to precision practice.

As an advanced subfield of machine learning and an indispensable backbone of health data science, deep learning provides a critical technical layer for the integrated analysis of multi-omics data [179–181]. Intelligent models based on region-based convolutional neural networks (RCNN) enable high-precision automated identification and classification of RBCs in microscopic images, effectively overcoming analytical challenges such as cell adhesion and overlap [182]. Recent studies have further established a low-cost, label-free strategy that integrates microfluidics with optimized deep learning for multi-class morphological classification of RBCs, allowing quantitative assessment of stored blood quality through morphological indices [183]. These approaches convert hard-to-quantify phenotypic features such as RBC morphology into digitized, model-ready data, filling the gap left by conventional multi-omics in the quantitative analysis of cellular morphological phenotypes [182]. By further coupling deep learning with microfluidic platforms [184], researchers can enrich the data dimensions for RSL phenotypic characterization, forming a robust closed loop of multidimensional feature data.

Nevertheless, such analytical pipelines remain unidirectional, lacking feedback loops for iterative experimental optimization. A bidirectional closed-loop architecture defined as OOC–AI virtual cell (OOC–AIVC) system has been proposed to mitigate this limitation; this framework realizes reciprocal calibration between microphysiological chips and multi-scale computational models, facilitating multi-modal data fusion and longitudinal individualized prediction of RSL progression [185].

Building upon the bidirectional calibration mechanism of the OOC–AIVC system, the deep convergence of microfluidic platforms, multi-omics technologies, and machine learning is expected to constitute the core technical system of a “digital twin blood bag”, enabling precise prediction and dynamic monitoring of the entire RBC storage cycle. The specific technical concept is detailed as follows. In the early stage of blood donation, a small number of RBCs are extracted from donor samples. On one hand, single-cell deformability distribution, membrane mechanical characteristics, and morphometric quantitative phase images are obtained via microfluidic chips, with digitized analysis of morphological and mechanical features completed by deep learning models. On the other hand, limited multi-omics data (genomics, metabolomics, proteomics, etc.) of the sample are collected synchronously, and multi-modal data fusion is performed on digitized morphological/mechanical features and multi-omics molecular signatures. A multi-modal machine learning model is trained using large-sample full-cycle data of RBC storage. Parallelized single-cell tracking algorithms will be embedded into the unified analytical pipeline to realize simultaneous profiling of hundreds of individual RBCs, greatly lifting the limited sample processing capacity of traditional chip observation workflows; meanwhile, the trained multi-modal model will generate universal standardized scoring cutoffs for RSL severity, eliminating inconsistent comparative results caused by the lack of unified quantitative evaluation criteria across different experimental batches. The model can not only predict traditional regulatory endpoints such as hemolysis rate and 24-h in vivo survival rate but also further forecast functional endpoints closer to clinical transfusion outcomes, including early splenic clearance ratio and magnitude of microcirculatory resistance elevation. Thereby, an “expected performance curve” for the unit is generated during blood product manufacturing, realizing early prediction of storage quality.

On a broader scale, massive chip readings and omics data from diverse regions and populations can be aggregated into a shared model, continuously enhancing the model’s adaptability to different races, diets, disease spectra, and storage practices. Ultimately, a cross-population and cross-system predictive engine will be formed, driving transfusion medicine toward full-chain precision management (Fig. 6B).

### Functional quality control for precision transfusion

Traditional quality control of RBC units in blood banks centers on storage duration, supplemented by a small number of biochemical indicators. It only enables basic safety screening and fails to precisely characterize storage-induced impairment of RBC deformability and its actual impact on microcirculatory perfusion. Thus, it is inadequate to meet the clinical transfusion needs of diverse recipients. Studies using microfluidic capillary network technologies—including the artificial microvascular network and multiplexed microcapillary network—have confirmed that these platforms exhibit markedly higher sensitivity to storage-induced decline of RBC deformability than conventional ektacytometry. They can not only identify functional differences of RBCs under distinct storage conditions at an early storage stage but also quantify capillary plugging-related metrics to accurately reflect the actual traversal capability of RBCs in physiological microcirculation, providing clinically relevant core indicators for the functional quality assessment of RBCs [186].

Driven by the high sensitivity and functional relevance of microfluidic technologies, the future quality control system of blood banks is expected to undergo a pivotal transformation from time-based management to function-based scoring, establishing functional quality control standards for RBCs centered on microvascular traversal capacity. Specifically, prior to clinical release, each unit of RBCs may undergo a standardized microfluidic testing panel for comprehensive detection and scoring of multidimensional functional indicators—including microvascular network perfusion rate, single-cell rigidity spectrum distribution, and capillary plugging risk—replacing the traditional single-evaluation model to achieve precise quantification of RBC functional status.

Industrialized pre-endothelialized disposable chip cartridges pre-coated with anti-fouling ECM will be developed to remove complex on-site cell culture steps and shorten total testing duration, lowering the professional operation threshold for routine blood bank staff. This functional quality control system will directly facilitate the implementation of precision transfusion matching, enabling risk-sensitive transfusion strategies tailored to the clinical characteristics of recipients. For microcirculation-vulnerable populations such as patients with cardiovascular and cerebrovascular diseases, premature infants, and critically ill patients in intensive care units, RBC units with high perfusion rate, low rigidity percentile, and low plugging risk in microfluidic tests may be preferentially selected to minimize the adverse effects of storage-damaged RBCs on the recipient’s microcirculation. For non-microcirculation-sensitive populations such as patients undergoing elective surgery, under the premise of meeting functional safety thresholds, blood bank inventory turnover efficiency and transfusion costs may be balanced to achieve optimal resource allocation (Fig. 6C).

### Microfluidic optimization of storage strategies

Microfluidic technologies exhibit great application potential in optimizing RBC storage systems by virtue of precise fluid manipulation, micro-volume sample detection, and automated regulation capabilities. Studies have confirmed that a semi-autonomous glucose delivery system based on microperistaltic pumps combined with Wi-Fi wireless control enables periodic bolus glucose supplementation in the modified AS-1N normoglycemic storage system, stably maintaining glucose concentration in the storage system at a physiological level (normoglycemic state). Compared with the traditional hyperglycemic storage solution (AS-1), this method can markedly reduce the RBC lysis rate and hemoglobin glycation damage, enhance the ATP release capacity of RBCs, and improve the deformation performance related to single-cell Young’s modulus, realizing the synchronous optimization of chemical and physical-mechanical properties of RBCs during storage [187] . This technical practice proves that active regulation of the RBC storage microenvironment via microfluidic technologies can effectively alleviate storage lesions and provide critical technical support for the precise design of storage strategies.

On the basis of microfluidic-mediated precise regulation of the storage microenvironment, future RBC storage systems can break through the passive storage mode of traditional blood bags and upgrade to integrated and programmable “microfluidic smart blood storage microreactors”. The blood storage system will be embedded with microfluidic channels, high-precision microperistaltic pumps, gas exchange membranes, and microsensor modules. This could enable dynamic and programmed adjustment of key parameters of the storage microenvironment using automated control algorithms. For example, in the early stage of storage, glucose supplementation rate can be precisely regulated through micropumps to maintain a physiological normoglycemic environment and inhibit non-enzymatic glycation; oxygen tension can be adjusted through gas exchange membranes to reduce oxidative stress damage. In the middle stage, intermittent and low-amplitude shear stress can be introduced and regulated to simulate the mechanical environment of in vivo microcirculation through microfluidic hydrodynamics, maintain the plasticity of RBC membrane–cytoskeleton, and delay cell stiffening. In the late stage of storage and before transfusion, an integrated microfluidic re-processing program can be started, where 3D porous structures or splenic sinusoid-mimetic micro-slits physically “polish” and screen the rigid RBC subpopulations produced during storage and efficient restoration of the physiological state of RBCs is realized with blood-gas equilibrium and ion concentration calibration through microfluidic channels. Thermoplastic injection molding will replace lab-scale soft lithography to realize standardized mass production of microreactor hardware, forming stable, replicable manufacturing pipelines; multi-parallel channel arrays will be integrated into each reactor unit to simultaneously process multiple independent blood unit samples, greatly elevating the screening efficiency for different storage additive formulations and dynamic storage regimens (Fig. 6D).

## Conclusion

Offering microscale biomimetic design, high-throughput single-cell analysis, and physiological shear environment simulation, microfluidic chip technology has opened a new avenue for addressing the quality control challenges of RSL and advancing the development of precision transfusion. It could enable paradigm shifts in this field from static population-based indicator detection in storage bags to physiological dynamic single-cell mechanical and functional characterization as well as microcirculatory systemic interaction research. The integrated application of LOC and OOC can not only precisely reveal the donor heterogeneity of RSL and fill the research gap between cellular phenotypes and in vivo microcirculatory effects but also establish a full-chain technical support system covering intelligent storage quality assessment, isolation of deteriorated cells, and storage strategy optimization, laying an important foundation for the transformation of transfusion medicine from empirical time-based management to functional precision matching.

The application of microfluidic chips in RSL quality control relies on the progress in ongoing technical iteration and clinical translation. Optimization of material biocompatibility, standardization of fabrication processes, and quantitative correlation between detection indicators and clinical outcomes remain core issues that need to be urgently addressed. In the future, the deep integration of this technology with multi-omics, machine learning, and synthetic biology, as well as the development and application of vascularized MOC, is expected to enable precise prediction and dynamic regulation of the entire RBC storage cycle, thereby promoting the establishment of functional quality control standards and the development of intelligent blood storage systems. This will continuously improve the safety and efficacy of clinical transfusion, and offer a powerful technical platform for research in related fields such as hematology and microcirculatory biology, demonstrating interdisciplinary scientific value and broad clinical application prospects.

## References

[1] D’Alessandro A. From omics technologies to personalized transfusion medicine. Expert Rev Proteomics. 2019;16(3):215–225. 30654673 10.1080/14789450.2019.1571917

[2] Yoshida T, Prudent M, D'Alessandro A. Red blood cell storage lesion: Causes and potential clinical consequences. Blood Transfus. 2019;17(1):27–52. 30653459 10.2450/2019.0217-18 PMC6343598

[3] D'Alessandro A, Kriebardis AG, Rinalducci S, Antonelou MH, Hansen KC, Papassideri IS, Zolla L. An update on red blood cell storage lesions, as gleaned through biochemistry and omics technologies. Transfusion. 2015;55(1):205–219. 25130459 10.1111/trf.12804

[4] Garraud O, Tariket S, Sut C, Haddad A, Aloui C, Chakroun T, Laradi S, Cognasse F. Transfusion as an inflammation hit: Knowns and unknowns. Front Immunol. 2016;7:534. 27965664 10.3389/fimmu.2016.00534 PMC5126107

[5] van Manen L, van Hezel ME, Boshuizen M, Straat M, de Man AM, Dekimpe C, Vanhoorelbeke K, van Bruggen R, Juffermans NP. Effect of red blood cell transfusion on inflammation, endothelial cell activation and coagulation in the critically ill. Vox Sang. 2022;117(1):64–70. 34196412 10.1111/vox.13125 PMC9291904

[6] Cluitmans JCA, Chokkalingam V, Janssen AM, Brock R, Huck WTS, Bosman GJ. Alterations in red blood cell deformability during storage: A microfluidic approach. Biomed Res Int. 2014;2014(1): Article 764268. 25295273 10.1155/2014/764268 PMC4176636

[7] Caughey MC, Francis RO, Karafin MS. New and emerging technologies for pretransfusion blood quality assessment: A state-of-the-art review. Transfusion. 2024;64(11):2196–2208. 39325509 10.1111/trf.18019 PMC11573642

[8] Grigorev GV, Lebedev AV, Wang X, Qian X, Maksimov GV, Lin L. Advances in microfluidics for single red blood cell analysis. Biosensors. 2023;13(1):117. 36671952 10.3390/bios13010117 PMC9856164

[9] Xu X, Zhang Q, Li M, Lin S, Liang S, Cai L, Zhu H, Su R, Yang C. Microfluidic single-cell multiomics analysis. View. 2023;4(1):20220034.

[10] Wang H, Chen K, Wang Z. Microfluidic single-cell technologies for immunotherapeutic target discovery: From design concepts to preclinical applications. View. 2026;7(1):20250065.

[11] Bosman G. Survival of red blood cells after transfusion: Processes and consequences. Front Physiol. 2013;4: Article 376. 24391593 10.3389/fphys.2013.00376 PMC3866658

[12] Lutz HU, Bogdanova A. Mechanisms tagging senescent red blood cells for clearance in healthy humans. Front Physiol. 2013;4: Article 387. 24399969 10.3389/fphys.2013.00387 PMC3872327

[13] Kriebardis AG, Antonelou MH, Stamoulis KE, Economou-Petersen E, Margaritis LH, Papassideri IS. Progressive oxidation of cytoskeletal proteins and accumulation of denatured hemoglobin in stored red cells. J Cell Mol Med. 2007;11(1):148–155. 17367509 10.1111/j.1582-4934.2007.00008.x PMC4401228

[14] Dinkla S, Novotný VMJ, Joosten I, Bosman GJCGM. Storage-induced changes in erythrocyte membrane proteins promote recognition by autoantibodies. PLOS ONE. 2012;7(8): Article e42250. 22879923 10.1371/journal.pone.0042250 PMC3411782

[15] Hess JR. Red cell changes during storage. Transfus Apher Sci. 2010;43(1):51–59. 20558107 10.1016/j.transci.2010.05.009

[16] Adams F, Bellairs G, Bird AR, Oguntibeju OO. Biochemical storage lesions occurring in nonirradiated and irradiated red blood cells: A brief review. Biomed Res Int. 2015;2015(1): Article 968302. 25710038 10.1155/2015/968302 PMC4325969

[17] Nemkov T, Stephenson D, Earley EJ, Keele GR, Hay A, Key A, Haiman ZB, Erickson C, Dzieciatkowska M, Reisz JA, et al. Biological and genetic determinants of glycolysis: Phosphofructokinase isoforms boost energy status of stored red blood cells and transfusion outcomes. Cell Metab. 2024;36(9):1979–1997.e13. 38964323 10.1016/j.cmet.2024.06.007 PMC11374506

[18] Jana S, Kassa T, Wood F, Hicks W, Alayash AI. Changes in hemoglobin oxidation and band 3 during blood storage impact oxygen sensing and mitochondrial bioenergetic pathways in the human pulmonary arterial endothelial cell model. Front Physiol. 2023;14:1278763. 37916221 10.3389/fphys.2023.1278763 PMC10617028

[19] Anastasiadi AT, Stamoulis K, Papageorgiou EG, Lelli V, Rinalducci S, Papassideri IS, Kriebardis AG, Antonelou MH, Tzounakas VL. The time-course linkage between hemolysis, redox, and metabolic parameters during red blood cell storage with or without uric acid and ascorbic acid supplementation. Front Aging. 2023;4:1161565. 37025499 10.3389/fragi.2023.1161565 PMC10072267

[20] Flatt JF, Bawazir WM, Bruce LJ. The involvement of cation leaks in the storage lesion of red blood cells. Front Physiol. 2014;5:00214. 10.3389/fphys.2014.00214 PMC4060409 24987374

[21] Antonelou MH, Kriebardis AG, Papassideri IS. Aging and death signalling in mature red cells: From basic science to transfusion practice. Blood Transfus. 2010;8(Suppl 3):s39–s47. 20606748 10.2450/2010.007S PMC2897187

[22] Rapido F, Brittenham GM, Bandyopadhyay S, La Carpia F, L’Acqua C, McMahon DJ, Rebbaa A, Wojczyk BS, Netterwald J, Wang H, et al. Prolonged red cell storage before transfusion increases extravascular hemolysis. J Clin Invest. 2017;127(1):375–382. 27941245 10.1172/JCI90837 PMC5199711

[23] Hod EA, Zhang N, Sokol SA, Wojczyk BS, Francis RO, Ansaldi D, Francis KP, Della-Latta P, Whittier S, Sheth S, et al. Transfusion of red blood cells after prolonged storage produces harmful effects that are mediated by iron and inflammation. Blood. 2010;115(21):4284–4292. 20299509 10.1182/blood-2009-10-245001 PMC2879099

[24] Flegel WA, Natanson C, Klein HG. Does prolonged storage of red blood cells cause harm? Br J Haematol. 2014;165(1):3–16. 24460532 10.1111/bjh.12747 PMC5515544

[25] Reilly M, Bruno CD, Prudencio TM, Ciccarelli N, Guerrelli D, Nair R, Ramadan M, Luban NL, Posnack NG. Potential consequences of the red blood cell storage lesion on cardiac electrophysiology. J Am Heart Assoc. 2020;9(21): Article e017748. 33086931 10.1161/JAHA.120.017748 PMC7763412

[26] Moroz V, Sherstyukova E, Kozlova E, Sergunova V. Storage time of filtered red blood cells and post-transfusion complications. Obs Reanimatol. 2021;17(1):69–82.

[27] Anastasiadi AT, Stamoulis K, Kriebardis AG, Tzounakas VL. Molecular modifications to mitigate oxidative stress and improve red blood cell storability. Front Physiol. 2024;15:1499308. 39539958 10.3389/fphys.2024.1499308 PMC11557539

[28] Lopes MG, Recktenwald SM, Simionato G, Eichler H, Wagner C, Quint S, Kaestner L. Big data in transfusion medicine and artificial intelligence analysis for red blood cell quality control. Transfus Med Hemother. 2023;50(3):163–173. 37408647 10.1159/000530458 PMC10319094

[29] Zhao D, Chen R, Jiang S, Xie W, Zhang K, Zhao J, Feng S, Bian S. Development and application of microfluidic sweat detection technology in mental health monitoring. View. 2025;6(3):20240088.

[30] Ramón-Azcón J, Rydosz A. Human organs-on-a-chip: Novel organ-on-a-chip techniques in medicine. Cambridge (MA): Academic Press; 2023.

[31] Azizipour N, Avazpour R, Rosenzweig DH, Sawan M, Ajji A. Evolution of biochip technology: A review from lab-on-a-chip to organ-on-a-chip. Micromachines. 2020;11(6):599. 32570945 10.3390/mi11060599 PMC7345732

[32] Gong X, Yang C, Peng J, Ding X, Yang H, Wang AG, Dzakah EE, Zhao B. Vascularized organoid-on-a-chip for centimeter-scale organoid cultivation. Bio-Des Manuf. 2025;8(3):410–422.

[33] Geng Y, Yu J. Progress in constructing functional coacervate systems using microfluidics. BMEMat. 2024;2(1): Article e12058.

[34] Lei KF. Microfluidic systems for diagnostic applications: A review. J Lab Autom. 2012;17(5):330–347. 22893635 10.1177/2211068212454853

[35] Conde JP, Madaboosi N, Soares RR, Fernandes JT, Novo P, Moulas G, Chu V. Lab-on-chip systems for integrated bioanalyses. Essays Biochem. 2016;60(1):121–131. 27365042 10.1042/EBC20150013 PMC4986467

[36] Li J, Zhang D, Meng Y, Chang Y, Wei W, Wu P, Peng L, Chang W, Wang W, Huang J, et al. Modeling fire-related smoke inhalation injury using the human lung-on-a-chip and organoid platform: Pathogenesis insights and therapeutic evaluation. View. 2025;6(3):20240114.

[37] Yin Q, Yang Z, Chong SW, Li J, Liu X, Vigolo D, Li JJ, Sheehy PA, Yong KT. Application of microfluidic technologies in veterinary science with a view toward development of animal-on-a-chip models. View. 2025;6(1):20240073.

[38] Wang J, Liang Z, Wang J, Li Z, Wang S, Wei Y, Xie X, Huang D. Microengineering the liver: Strategies for constructing functional liver-on-a-chip devices. Exploration. 2026;6(2): Article 70137. 42016750 10.1002/exp2.70137 PMC13094531

[39] Qian K, Wang Y, Li R, Zhuang M, Zhao Z, Meng C, Zhou J, Wang W, Sun Y. Application and prospects of organoid-on-a-chip in research on the intestinal mucosal barrier. Burns Trauma. 2026; Article tkag029. 42544182 10.1093/burnst/tkag029 PMC13429053

[40] Zheng F, Fu F, Cheng Y, Wang C, Zhao Y, Gu Z. Organ-on-a-chip systems: Microengineering to biomimic living systems. Small. 2016;12(17):2253–2282. 26901595 10.1002/smll.201503208

[41] Esch EW, Bahinski A, Huh D. Organs-on-chips at the frontiers of drug discovery. Nat Rev Drug Discov. 2015;14(4):248–260. 25792263 10.1038/nrd4539 PMC4826389

[42] De Rudder C, Greenhalgh K, Baginska J, Martin-Gallausiaux C, Grandmougin L, Villette R, Lucchetti M, Hansen B, Ostaszewski M, Petrov VA, et al. Immunohumix: A personalizable gut-on-chip model for unraveling human microbiome–immune interactions. View. 2026; Article 20250213.

[43] Zhong Q, Ding H, Gao B, He Z, Gu Z. Advances of microfluidics in biomedical engineering. Adv Mater Technol. 2019;4(6):1800663.

[44] Leung CM, De Haan P, Ronaldson-Bouchard K, Kim GA, Ko J, Rho HS, Chen Z, Habibovic P, Jeon NL, Takayama S, et al. A guide to the organ-on-a-chip. Nat Rev Methods Primers. 2022;2(1):33.

[45] Testa M, Palumbo L, La Rosa M, Girgenti A, Palumbo FS, Picone P, Lopresti F, Nuzzo D, La Carruba V. A thermoplastic microfluidic platform with integrated electrospun scaffolds for assessing brain cell-specific drug selectivity. View. 2026; Article 20250211.

[46] Haeberle S, Zengerle R. Microfluidic platforms for lab-on-a-chip applications. Lab Chip. 2007;7(9):1094–1110. 17713606 10.1039/b706364b

[47] Khosla NK, Lesinski JM, Colombo M, Bezinge L, deMello AJ, Richards DA. Simplifying the complex: Accessible microfluidic solutions for contemporary processes within in vitro diagnostics. Lab Chip. 2022;22(18):3340–3360. 35984715 10.1039/d2lc00609j PMC9469643

[48] Mark D, Haeberle S, Roth G, von Stetten F, Zengerle R. Microfluidic lab-on-a-chip platforms: Requirements, characteristics and applications. Chem Soc Rev. 2010;39(3):1153–1182. 20179830 10.1039/b820557b

[49] Nguyen N-T, Hejazian M, Ooi CH, Kashaninejad N. Recent advances and future perspectives on microfluidic liquid handling. Micromachines. 2017;8(6):186.

[50] Iakovlev AP, Erofeev AS, Gorelkin PV. Novel pumping methods for microfluidic devices: A comprehensive review. Biosensors. 2022;12(11):956. 36354465 10.3390/bios12110956 PMC9688261

[51] Sin MLY, Gao J, Liao JC, Wong PK. System integration—A major step toward lab on a chip. J Biol Eng. 2011;5(1):6. 21612614 10.1186/1754-1611-5-6 PMC3117764

[52] Ancelmo HC, Schuster da Silva S, Kuchenbecker HF, Canniatti Brazaca L, Blanes L. Microfluidic flow control strategies for organ-on-a-chip devices: A critical review. Discov Electron. 2026;3(1):52.

[53] Strohmeier O, Keller M, Schwemmer F, Zehnle S, Mark D, von Stetten F, Zengerle R, Paust N. Centrifugal microfluidic platforms: Advanced unit operations and applications. Chem Soc Rev. 2015;44(17):6187–6229. 26035697 10.1039/c4cs00371c

[54] Madou M, Zoval J, Jia G, Kido H, Kim J, Kim N. Lab on a CD. Annu Rev Biomed Eng. 2006;8(1):601–628. 16834568 10.1146/annurev.bioeng.8.061505.095758

[55] Smith S, Mager D, Perebikovsky A, Shamloo E, Kinahan D, Mishra R, Torres Delgado SM, Kido H, Saha S, Ducrée J, et al. CD-based microfluidics for primary care in extreme point-of-care settings. Micromachines. 2016;7(2):22. 30407395 10.3390/mi7020022 PMC6190444

[56] Hess JF, Zehnle S, Juelg P, Hutzenlaub T, Zengerle R, Paust N. Review on pneumatic operations in centrifugal microfluidics. Lab Chip. 2019;19(22):3745–3770. 31596297 10.1039/c9lc00441f

[57] Bousse L, Cohen C, Nikiforov T, Chow A, Kopf-Sill AR, Dubrow R, Parce JW. Electrokinetically controlled microfluidic analysis systems. Annu Rev Biophys. 2000;29(1):155–181. 10.1146/annurev.biophys.29.1.155 10940246

[58] Wang X, Cheng C, Wang S, Liu S. Electroosmotic pumps and their applications in microfluidic systems. Microfluid Nanofluid. 2009;6(2):145–162. 20126306 10.1007/s10404-008-0399-9 PMC2756694

[59] Suea-Ngam A, Howes PD, Srisa-Art M, deMello AJ. Droplet microfluidics: From proof-of-concept to real-world utility? Chem Commun. 2019;55(67):9895–9903. 10.1039/c9cc04750f 31334541

[60] Teh S-Y, Lin R, Hung L-H, Lee AP. Droplet microfluidics. Lab Chip. 2008;8(2):198–220. 18231657 10.1039/b715524g

[61] Shi N, Mohibullah M, Easley CJ. Active flow control and dynamic analysis in droplet microfluidics. Annu Rev Anal Chem. 2021;14:133–153. 10.1146/annurev-anchem-122120-042627 PMC8956363 33979546

[62] Rosenfeld L, Lin T, Derda R, Tang SKY. Review and analysis of performance metrics of droplet microfluidics systems. Microfluid Nanofluid. 2014;16(5):921–939.

[63] Fair RB. Digital microfluidics: Is a true lab-on-a-chip possible? Microfluid Nanofluid. 2007;3(3):245–281.

[64] Choi K, Ng AHC, Fobel R, Wheeler AR. Digital microfluidics. Annu Rev Anal Chem. 2012;5:413–440. 10.1146/annurev-anchem-062011-143028 22524226

[65] Samiei E, Tabrizian M, Hoorfar M. A review of digital microfluidics as portable platforms for lab-on a-chip applications. Lab Chip. 2016;16(13):2376–2396. 27272540 10.1039/c6lc00387g

[66] Xu X, Cai L, Liang S, Zhang Q, Lin S, Li M, Yang Q, Li C, Han Z, Yang C. Digital microfluidics for biological analysis and applications. Lab Chip. 2023;23(5):1169–1191. 36644972 10.1039/d2lc00756h

[67] Yang C, Gan X, Zeng Y, Xu Z, Xu L, Hu C, Ma H, Chai B, Hu S, Chai Y. Advanced design and applications of digital microfluidics in biomedical fields: An update of recent progress. Biosens Bioelectron. 2023;242: Article 115723. 37832347 10.1016/j.bios.2023.115723

[68] Zhang Y, Liu Y. Advances in integrated digital microfluidic platforms for point-of-care diagnosis: A review. Sens Diagn. 2022;1(4):648–672.

[69] Sun J, Wu Z, Li J, Shang L, Zhao Y, Li L. Organs-on-a-chip recapitulating the gut-islets axis for endocrine hormone secretion regulator evaluation. Research. 2025;8:0923. 41079673 10.34133/research.0923 PMC12508524

[70] Moses SR, Adorno JJ, Palmer AF, Song JW. Vessel-on-a-chip models for studying microvascular physiology, transport, and function in vitro. Am J Phys Cell Phys. 2021;320(1):C92–C105. 10.1152/ajpcell.00355.2020 PMC7846973 33176110

[71] Tan SY, Leung Z, Wu AR. Recreating physiological environments in vitro: Design rules for microfluidic-based vascularized tissue constructs. Small. 2020;16(9):1905055. 10.1002/smll.201905055 31913580

[72] You L, Chen Y, Zhang Z, Wang Y, Gu Z, Shen L, Ge J. The flow framework: A panvascular-on-a-chip platform to model systemic disease and guide panvascular interventional device suitcordance. Sci Bull. 2026;71(3):654–670. 10.1016/j.scib.2025.12.051 41535150

[73] Myers DR, Lam WA. Vascularized microfluidics and their untapped potential for discovery in diseases of the microvasculature. Annu Rev Biomed Eng. 2021;23:407–432. 33863238 10.1146/annurev-bioeng-091520-025358 PMC8785195

[74] Dellaquila A, Le Bao C, Letourneur D, Simon-Yarza T. In vitro strategies to vascularize 3D physiologically relevant models. Adv Sci. 2021;8(19): Article e2100798. 10.1002/advs.202100798 PMC8498873 34351702

[75] Zhang B, Montgomery M, Chamberlain MD, Ogawa S, Korolj A, Pahnke A, Wells LA, Massé S, Kim J, Reis L, et al. Biodegradable scaffold with built-in vasculature for organ-on-a-chip engineering and direct surgical anastomosis. Nat Mater. 2016;15(6):669–678. 26950595 10.1038/nmat4570 PMC4879054

[76] Vajda J, Milojević M, Maver U, Vihar B. Microvascular tissue engineering—A review. Biomedicine. 2021;9(6):589. 10.3390/biomedicines9060589 PMC8224375 34064101

[77] Catarino SO, Rodrigues RO, Pinho D, Miranda JM, Minas G, Lima R. Blood cells separation and sorting techniques of passive microfluidic devices: From fabrication to applications. Micromachines. 2019;10(9):593. 31510012 10.3390/mi10090593 PMC6780402

[78] Dalili A, Samiei E, Hoorfar M. A review of sorting, separation and isolation of cells and microbeads for biomedical applications: Microfluidic approaches. Analyst. 2018;144(1):87–113. 30402633 10.1039/c8an01061g

[79] Hesh CA, Qiu Y, Lam WA. Vascularized microfluidics and the blood–endothelium interface. Micromachines. 2020;11(1):18. 10.3390/mi11010018 PMC7019435 31878018

[80] Niculescu A-G, Chircov C, Bîrcă AC, Grumezescu AM. Fabrication and applications of microfluidic devices: A review. Int J Mol Sci. 2021;22(4):2011. 33670545 10.3390/ijms22042011 PMC7921936

[81] Pečar B, Resnik D, Možek M, Vrtačnik D. Microfluidics: A review. Informacije MIDEM. 2021;51(1):3–23.

[82] Alrifaiy A, Lindahl OA, Ramser K. Polymer-based microfluidic devices for pharmacy, biology and tissue engineering. Polymers. 2012;4(3):1349–1398.

[83] Halldorsson S, Lucumi E, Gómez-Sjöberg R, Fleming RMT. Advantages and challenges of microfluidic cell culture in polydimethylsiloxane devices. Biosens Bioelectron. 2015;63:218–231. 25105943 10.1016/j.bios.2014.07.029

[84] Szydzik C, Brazilek RJ, Nesbitt WS. A review of design considerations for hemocompatibility within microfluidic systems. Semin Thromb Hemost. 2020;46(5):622–636. 32604421 10.1055/s-0040-1710340

[85] Nahak BK, Mishra A, Preetam S, Tiwari A. Advances in organ-on-a-chip materials and devices. ACS Appl Bio Mater. 2022;5(8):3576–3607. 10.1021/acsabm.2c00041 35839513

[86] Li Z, Hui J, Yang P, Mao H. Microfluidic organ-on-a-chip system for disease modeling and drug development. Biosensors. 2022;12(6):370. 35735518 10.3390/bios12060370 PMC9220862

[87] Shou X, Yu Y, Wu D, Lu P, Zhao M, Zhao Y. Dynamic tumor immunology-on-a-chip for peripheral blood-derived tumor-reactive t cell expansion. Research. 2025;8:0639. 40123996 10.34133/research.0639 PMC11927211

[88] Zhang X-l, Hu M-n, Li Y-n, Wang Y, Rong Z, Ma J. Effect of electroacupuncture on the MMP-2/9 pathway mediated blood-brain barrier permeability in mice with Parkinson's disease: 电针对帕金森病小鼠 MMP-2/−9 通路介导的血脑屏障通透性的影响. World J Acupunct Moxibustion. 2025;35(1):58–65.

[89] Guan Z, Liu Q, Wang Y, Xiao F, Zhao G. Relationship between tight junctions of the BBB and astrocyte connective function in epilepsy: Albumin and astrocyte activation. Med Plus. 2024;1(3): Article 100047.

[90] Du Y, Li Z, Li X, Xie J. Advances in in vitro blood–brain barrier models: Integrating bioprinting with microfluidic chips for compound evaluation. Int J Bioprint. 2025;11(5):68.

[91] Puryear JR III, Yoon J-K, Kim Y. Advanced fabrication techniques of microengineered physiological systems. Micromachines. 2020;11(8):730. 32731495 10.3390/mi11080730 PMC7464561

[92] Cao UM, Zhang Y, Chen J, Sayson D, Pillai S, Tran SD. Microfluidic organ-on-a-chip: A guide to biomaterial choice and fabrication. Int J Mol Sci. 2023;24(4):3232. 36834645 10.3390/ijms24043232 PMC9966054

[93] Ingber DE. Human organs-on-chips for disease modelling, drug development and personalized medicine. Nat Rev Genet. 2022;23(8):467–491. 35338360 10.1038/s41576-022-00466-9 PMC8951665

[94] Shang Y, Xu D, Sun L, Zhao Y, Sun L. A biomimetic optical cardiac fibrosis-on-a-chip for high-throughput anti-fibrotic drug screening. Research. 2024;7:0471. 39268502 10.34133/research.0471 PMC11391215

[95] Liu K-K, Wu R-G, Chuang Y-J, Khoo HS, Huang S-H, Tseng F-G. Microfluidic systems for biosensing. Sensors. 2010;10(7):6623–6661. 22163570 10.3390/s100706623 PMC3231127

[96] Ferreira M, Carvalho V, Ribeiro J, Lima RA, Teixeira S, Pinho D. Advances in microfluidic systems and numerical modeling in biomedical applications: A review. Micromachines. 2024;15(7):873. 39064385 10.3390/mi15070873 PMC11279158

[97] Savitri G. Advancement in generation and application of microfluidic chip technology. Int J Pharm Sci Nanotechnol. 2024;17(2):7277–7298.

[98] Olaizola-Rodrigo C, Bayona C, Oliván S, Monge R. A review of organ-on-chip fabrication methods: From early developments to overcoming inert barriers. iScience. 2025;28(12):113992. 41377670 10.1016/j.isci.2025.113992 PMC12689227

[99] Zhou C, Li Z, Xu J, Yu D, Xuan L, Wang X. Multilayered microfluidic platform for three-dimensional vascularized organ-on-a-chip applications. Bio-Des Manuf. 2025;8(6):930–947.

[100] Hou Y, Ziakas G, Hopkins T, Wang W, HRC S, Knight MM. Human coronary artery organ-chip with circulating immune cells recapitulates anti-inflammatory effect of pulsatile wall strain. Interdiscip Med. 2026;4(3): Article e70114.

[101] Kaya B, Yesil-Celiktas O. Ionic liquid-based transparent membrane-coupled human lung epithelium-on-a-chip demonstrating pm0.5 pollution effect under breathing mechanostress. Bio Des Manuf. 2024;7(5):624–636.

[102] Kim JJ, Bae M, Kim J, Cho D-W. Application of biomaterial-based three-dimensional bioprinting for organ-on-a-chip fabrication. Int J Bioprint. 2024;10(1):1972.

[103] Li S, Liu X, Zhang L, Wang Q. Integrated applications of microfluidics, organoids, and 3d bioprinting in in vitro 3d biomimetic models. Int J Bioprint. 2025;11(3):115–153.

[104] Bao W, Liu J, Du C, Liu S, Liu J, Zhu Z, Zhou L, Li ZA, Huang W, Lei Y. The role of 3d printing in skeletal muscle-on-a-chip models: Current applications and future potential. Mater Today Bio. 2025;34: Article 102222. 10.1016/j.mtbio.2025.102222 PMC12408411 40917517

[105] Kong B, Zhao Y. 3d bioprinting for biomedical applications. BME Front. 2023;4:0010. 37849677 10.34133/bmef.0010 PMC10521671

[106] Zhu X, Li Y, Long H, Wu Y, Sun N, Li S, Liu Y, Xie H, Bao J. Micro pattern-based 3d cell culture platform: An overview of technologies and applications. Exploration. 2026;6(2): Article 20240469. 42016763 10.1002/EXP.20240469 PMC13094535

[107] Shi Y, Han X, Zhang Z, Xu J, Liu G. Liver organoids: From 3d printing to biomedical applications. BMEMat. 2025;3(2): Article e12129.

[108] González-Duque MI, Breuninger A, Leis F, Michaud JB, Sivakumar S, Pautu V, Jaconi ME, Jobin M, Roux A. Tissue engineering in vitro leaflet- and 3-dimensional printing-based implant prototypes for infant mitral valve. BME Front. 2025;6:0159. 40777733 10.34133/bmef.0159 PMC12329791

[109] Wang Y, Gao Y, Pan Y, Zhou D, Liu Y, Yin Y, Yang J, Wang Y, Song Y. Emerging trends in organ-on-a-chip systems for drug screening. Acta Pharm Sin B. 2023;13(6):2483–2509. 37425038 10.1016/j.apsb.2023.02.006 PMC10326261

[110] Pradhan S, Banda OA, Farino CJ, Sperduto JL, Keller KA, Taitano R, Slater JH. Biofabrication strategies and engineered in vitro systems for vascular mechanobiology. Adv Healthc Mater. 2020;9(8): Article e1901255. 32100473 10.1002/adhm.201901255 PMC8579513

[111] Cheng S, Wei J, Liu S, Liu J, Luo X, Lan Y, Dong M, Zhou L, Huang W, Zhao C, et al. Precision and customization in regenerative medicine: The role of coaxial 3d printing. Biomed Technol. 2025;12: Article 100115.

[112] Chae S, Ha D-H, Lee H. 3d bioprinting strategy for engineering vascularized tissue models. Int J Bioprint. 2023;9(5):748. 37502273 10.18063/ijb.748 PMC10370342

[113] Seymour AJ, Westerfield AD, Cornelius VC, Skylar-Scott MA, Heilshorn SC. Bioprinted microvasculature: Progressing from structure to function. Biofabrication. 2022;14(2): Article 022002. 10.1088/1758-5090/ac4fb5 PMC8988885 35086069

[114] Kim S, Kim W, Lim S, Jeon JS. Vasculature-on-a-chip for in vitro disease models. Bioengineering. 2017;4(1):8. 28952486 10.3390/bioengineering4010008 PMC5590435

[115] Hu C, Chen Y, Tan MJA, Ren K, Wu H. Microfluidic technologies for vasculature biomimicry. Analyst. 2019;144(15):4461–4471. 31162494 10.1039/c9an00421a

[116] Galpayage Dona KNU, Hale JF, Salako T, Anandanatarajan A, Tran KA, DeOre BJ, Galie PA, Ramirez SH, Andrews AM. The use of tissue engineering to fabricate perfusable 3d brain microvessels in vitro. Front Physiol. 2021;12: Article 715431. 34531761 10.3389/fphys.2021.715431 PMC8438211

[117] Henderson AR, Choi H, Lee E. Blood and lymphatic vasculatures on-chip platforms and their applications for organ-specific in vitro modeling. Micromachines. 2020;11(2):147. 32013154 10.3390/mi11020147 PMC7074693

[118] Im G-B, Lin R-Z. Bioengineering for vascularization: Trends and directions of photocrosslinkable gelatin methacrylate hydrogels. Front Bioeng Biotechnol. 2022;10:1053491. 36466323 10.3389/fbioe.2022.1053491 PMC9713639

[119] Xie R, Zheng W, Guan L, Ai Y, Liang Q. Engineering of hydrogel materials with perfusable microchannels for building vascularized tissues. Small. 2020;16(15): Article e1902838. 31559675 10.1002/smll.201902838

[120] Liu J, Zhu M, Bai N, Huang N, Zhang W, Yang Z, Wang Y. Microfluidic perfusable pathological vasculature for atherosclerosis drug screening. Research. 2025;8:0902. 40979559 10.34133/research.0902 PMC12446761

[121] Morais AS, Mendes M, Cordeiro MA, Sousa JJ, Pais AC, Mihăilă SM, Vitorino C. Organ-on-a-chip: Ubi sumus? Fundamentals and design aspects. Pharmaceutics. 2024;16(5):615. 38794277 10.3390/pharmaceutics16050615 PMC11124787

[122] Su Y, Chen Y, Wei G, Shi Y. Organoid-based novel technology for antitumor drug screening. View. 2025;6(6):20250107.

[123] Zhao N, Pessell AF, Zhu N, Searson PC. Tissue-engineered microvessels: A review of current engineering strategies and applications. Adv Healthc Mater. 2024;13(21): Article e2303419. 38686434 10.1002/adhm.202303419 PMC11338730

[124] Shin YJ, Safina D, Zheng Y, Levenberg S. Microvascularization in 3d human engineered tissue and organoids. Annu Rev Biomed Eng. 2025;27(1):473–498. 40310885 10.1146/annurev-bioeng-103023-115236

[125] Xu Z, Zheng Y, Wang X, Shehata N, Wang C, Sun Y. Stiffness increase of red blood cells during storage. Microsyst Nanoeng. 2018;4(1):17103.

[126] Isiksacan Z, D’Alessandro A, Wolf SM, McKenna DH, Tessier SN, Kucukal E, Gokaltun AA, William N, Sandlin RD, Bischof J, et al. Assessment of stored red blood cells through lab-on-a-chip technologies for precision transfusion medicine. Proc Natl Acad Sci USA. 2023;120(32): Article e2115616120. 37494421 10.1073/pnas.2115616120 PMC10410732

[127] Acker JP, Marks DC, Sheffield WP. Quality assessment of established and emerging blood components for transfusion. J Blood Transfus. 2016;2016(1): Article 4860284. 28070448 10.1155/2016/4860284 PMC5192317

[128] Kim KB, Chun H, Kim HC, Chung TD. Red blood cell quantification microfluidic chip using polyelectrolytic gel electrodes. Electrophoresis. 2009;30(9):1464–1469. 19340832 10.1002/elps.200800448

[129] Matthews K, Myrand-Lapierre M-E, Ang RR, Duffy SP, Scott MD, Ma H. Microfluidic deformability analysis of the red cell storage lesion. J Biomech. 2015;48(15):4065–4072. 26477408 10.1016/j.jbiomech.2015.10.002

[130] Wang Y, You G, Chen P, Li J, Chen G, Wang B, Li P, Han D, Zhou H, Zhao L. The mechanical properties of stored red blood cells measured by a convenient microfluidic approach combining with mathematic model. Biomicrofluidics. 2016;10(2): Article 024104. 27014397 10.1063/1.4943861 PMC4788599

[131] Recktenwald SM, Lopes MGM, Peter S, Hof S, Simionato G, Peikert K, Hermann A, Danek A, van Bentum K, Eichler H, et al. Erysense, a lab-on-a-chip-based point-of-care device to evaluate red blood cell flow properties with multiple clinical applications. Front Physiol. 2022;13: Article 884690. 35574449 10.3389/fphys.2022.884690 PMC9091344

[132] Kajitani K, Ohtani T, Higuchi R, Chimura M, Sera F, Tsai C-HD, Ueda Y, Nishimura JI, Sakata Y. An on-chip deformability checker demonstrates that the severity of iron deficiency is associated with increased deformability of red blood cells. Sci Rep. 2025;15(1):19994. 40481296 10.1038/s41598-025-05483-2 PMC12144279

[133] Park HS, Eldridge WJ, Yang W-H, Crose M, Ceballos S, Roback JD, Chi JT, Wax A. Quantitative phase imaging of erythrocytes under microfluidic constriction in a high refractive index medium reveals water content changes. Microsyst Nanoeng. 2019;5(1):63. 31814994 10.1038/s41378-019-0113-y PMC6885519

[134] Guruprasad P, Mannino RG, Caruso C, Zhang H, Josephson CD, Roback JD, Lam WA. Integrated automated particle tracking microfluidic enables high-throughput cell deformability cytometry for red cell disorders. Am J Hematol. 2019;94(2):189–199. 30417938 10.1002/ajh.25345 PMC7007699

[135] Kwan JM, Guo Q, Kyluik-Price DL, Ma H, Scott MD. Microfluidic analysis of cellular deformability of normal and oxidatively damaged red blood cells. Am J Hematol. 2013;88(8):682–689. 23674388 10.1002/ajh.23476

[136] Islamzada E, Matthews K, Guo Q, Santoso AT, Duffy SP, Scott MD, Ma H. Deformability based sorting of stored red blood cells reveals donor-dependent aging curves. Lab Chip. 2020;20(2):226–235. 31796943 10.1039/c9lc01058k

[137] Islamzada E, Matthews K, Lamoureux ES, Duffy SP, Scott MD, Ma H. Degradation of red blood cell deformability during cold storage in blood bags. EJHaem. 2022;3(1):63–71. 35846223 10.1002/jha2.343 PMC9176030

[138] Huang S, Hou HW, Kanias T, Sertorio JT, Chen H, Sinchar D, Gladwin MT, Han J. Towards microfluidic-based depletion of stiff and fragile human red cells that accumulate during blood storage. Lab Chip. 2015;15(2):448–458. 25406942 10.1039/c4lc00768a PMC4268274

[139] Song M, Anderson CC, Sridhar N, Reisz JA, Akh L, Gao Y, D'alessandro A, Ding X. Surface acoustic wave hemolysis assay for evaluating stored red blood cells. Lab Chip. 2026;26(1):40–53. 41251214 10.1039/d5lc00652j PMC12624846

[140] Islamzada E, Matthews K, Lamoureux E, Duffy SP, Scott MD, Ma H. Blood unit segments accurately represent the biophysical properties of red blood cells in blood bags but not hemolysis. Transfusion. 2022;62(2):448–456. 34877683 10.1111/trf.16757

[141] Robidoux J, Laforce-Lavoie A, Charette SJ, Shevkoplyas SS, Yoshida T, Lewin A, Brouard D. Development of a flow standard to enable highly reproducible measurements of deformability of stored red blood cells in a microfluidic device. Transfusion. 2020;60(5):1032–1041. 32237236 10.1111/trf.15770 PMC9701565

[142] Chen Y-C, Chen G-Y, Lin Y-C, Wang G-J. A lab-on-a-chip capillary network for red blood cell hydrodynamics. Microfluid Nanofluid. 2010;9(2):585–591.

[143] Stauber H, Waisman D, Korin N, Sznitman J. Red blood cell dynamics in biomimetic microfluidic networks of pulmonary alveolar capillaries. Biomicrofluidics. 2017;11(1): Article 014103. 28090238 10.1063/1.4973930 PMC5234697

[144] Man Y, Kucukal E, An R, Watson QD, Bosch J, Zimmerman PA, Little JA, Gurkan UA. Microfluidic assessment of red blood cell mediated microvascular occlusion. Lab Chip. 2020;20(12):2086–2099. 32427268 10.1039/d0lc00112k PMC7473457

[145] Li M, Zhang L, Ullah Z, Zhang Y, Wei S, Huang L, Guo B. Artificial intelligence-assisted theranostics for brain tumors: Advancements and future perspectives. View. 2025;6(6): Article 20250119.

[146] Sun H, Wang J. Computational biomedical imaging: Ai innovations and pitfalls. Med Plus. 2025;2(2): Article 100081.

[147] Praljak N, Iram S, Goreke U, Singh G, Hill A, Gurkan UA, Hinczewski M. Integrating deep learning with microfluidics for biophysical classification of sickle red blood cells adhered to laminin. PLOS Comput Biol. 2021;17(11): Article e1008946. 34843453 10.1371/journal.pcbi.1008946 PMC8659663

[148] Doan M, Sebastian JA, Caicedo JC, Siegert S, Roch A, Turner TR, Mykhailova O, Pinto RN, McQuin C, Goodman A, et al. Objective assessment of stored blood quality by deep learning. Proc Natl Acad Sci USA. 2020;117(35):21381–21390. 32839303 10.1073/pnas.2001227117 PMC7474613

[149] Lamoureux ES, Islamzada E, Wiens MVJ, Matthews K, Duffy SP, Ma H. Assessing red blood cell deformability from microscopy images using deep learning. Lab Chip. 2021;22(1):26–39. 34874395 10.1039/d1lc01006a

[150] Fay ME, Oshinowo O, Iffrig E, Fibben KS, Caruso C, Hansen S, Musick JO, Valdez JM, Azer SS, Mannino RG, et al. Iclots: Open-source, artificial intelligence-enabled software for analyses of blood cells in microfluidic and microscopy-based assays. Nat Commun. 2023;14(1):5022. 37596311 10.1038/s41467-023-40522-4 PMC10439163

[151] Tsvirkun D, Grichine A, Duperray A, Misbah C, Bureau L. Microvasculature on a chip: Study of the endothelial surface layer and the flow structure of red blood cells. Sci Rep. 2017;7(1):45036. 28338083 10.1038/srep45036 PMC5364477

[152] Qiu Y, Ahn B, Sakurai Y, Hansen CE, Tran R, Mimche PN, Mannino RG, Ciciliano JC, Lamb TJ, Joiner CH, et al. Microvasculature-on-a-chip for the long-term study of endothelial barrier dysfunction and microvascular obstruction in disease. Nat Biomed Eng. 2018;2(6):453–463. 30533277 10.1038/s41551-018-0224-z PMC6286070

[153] Semenov AN, Lugovtsov AE, Ermolinskiy PB, Priezzhev AV. Laser aggregometry assessment of blood microrheology in a slit fluidic channel covered with endothelial cells. J Biophotonics. 2024;17(12): Article e202400379. 39389583 10.1002/jbio.202400379 PMC11614562

[154] Kucukal E, Ilich A, Key NS, Little JA, Gurkan UA. Red blood cell adhesion to heme-activated endothelial cells reflects clinical phenotype in sickle cell disease. Am J Hematol. 2018;93(8):1050–1060. 10.1002/ajh.25159 PMC6295270 29905377

[155] Kucukal E, Wolfe A, Kocevar R, Nayak LV, Sowemimo-Coker S, Zak J, Omert L, Gurkan UA. Hypoxic storage of red blood cells: Assessment of adhesion properties using a standardized endothelium-on-a-chip microfluidic platform. Blood. 2021;138(Supplement 1):1072.

[156] Mannino RG, Myers DR, Ahn B, Wang Y, Margo R, Gole H, Lin AS, Guldberg RE, Giddens DP, Timmins LH, et al. Do-it-yourself in vitro vasculature that recapitulates in vivo geometries for investigating endothelial-blood cell interactions. Sci Rep. 2015;5(1):12401. 26202603 10.1038/srep12401 PMC4894411

[157] Carden MA, Fay ME, Lu X, Mannino RG, Sakurai Y, Ciciliano JC, Hansen CE, Chonat S, Joiner CH, Wood DK, et al. Extracellular fluid tonicity impacts sickle red blood cell deformability and adhesion. Blood. 2017;130(24):2654–2663. 28978568 10.1182/blood-2017-04-780635 PMC5731085

[158] Wulftange W, Kucukal E, Man Y, An R, Monchamp K, Olayiwola O, Little JA, Key NS, Gurkan UA. Thrombin-induced endothelial cell damage is mitigated by human anti-thrombin iii in a microfluidic device. Blood. 2020;136(Supplement 1):24–25.32430494

[159] Tsai M, Kita A, Leach J, Rounsevell R, Huang JN, Moake J, Ware RE, Fletcher DA, Lam WA. In vitro modeling of the microvascular occlusion and thrombosis that occur in hematologic diseases using microfluidic technology. J Clin Invest. 2012;122(1):408–418. 22156199 10.1172/JCI58753 PMC3248292

[160] Qiu Y, Lin J, Wang A, Fang Z, Sakurai Y, Choi H, Williams EK, Hardy ET, Maher K, Coskun AF, et al. Clinically relevant clot resolution via a thromboinflammation-on-a-chip. Nature. 2025;641(8065):1298–1308. 40175551 10.1038/s41586-025-08804-7 PMC13138053

[161] Zheng Y, Chen J, Craven M, Choi NW, Totorica S, Diaz-Santana A, Kermani P, Hempstead B, Fischbach-Teschl C, López JA, et al. In vitro microvessels for the study of angiogenesis and thrombosis. Proc Natl Acad Sci USA. 2012;109(24):9342–9347. 22645376 10.1073/pnas.1201240109 PMC3386137

[162] Zheng Y, Chen J, López JA. Flow-driven assembly of vwf fibres and webs in in vitro microvessels. Nat Commun. 2015;6(1):7858. 26223854 10.1038/ncomms8858 PMC4522708

[163] Manz XD, Albers HJ, Symersky P, Aman J, van der Meer AD, Bogaard HJ, Szulcek R. In vitro microfluidic disease model to study whole blood-endothelial interactions and blood clot dynamics in real-time. J Vis Exp. 2020;(159): Article e61068. 10.3791/61068 32510519

[164] Jain A, van der Meer AD, Papa A-L, Barrile R, Lai A, Schlechter BL, Otieno MA, Louden CS, Hamilton GA, Michelson AD, et al. Assessment of whole blood thrombosis in a microfluidic device lined by fixed human endothelium. Biomed Microdevices. 2016;18(4):73. 27464497 10.1007/s10544-016-0095-6 PMC4963439

[165] Middelkamp HHT, Weener HJ, Gensheimer T, Vermeul K, de Heus LE, Albers HJ, Van den Berg A, Van Der Meer AD. Embedded macrophages induce intravascular coagulation in 3d blood vessel-on-chip. Biomed Microdevices. 2023;26(1):2. 38085384 10.1007/s10544-023-00684-w PMC10716057

[166] Chen SW, Blazeski A, Zhang S, Shelton SE, Offeddu GS, Kamm RD. Development of a perfusable, hierarchical microvasculature-on-a-chip model. Lab Chip. 2023;23(20):4552–4564. 37771308 10.1039/d3lc00512g PMC10563829

[167] Li ZA, Tuan RS. Towards establishing human body-on-a-chip systems. Stem Cell Res Ther. 2022;13(1):431. 35987699 10.1186/s13287-022-03130-5 PMC9392934

[168] Zhou Z, Liu J, Xiong T, Liu Y, Tuan RS, Li ZA. Engineering innervated musculoskeletal tissues for regenerative orthopedics and disease modeling. Small. 2024;20(23): Article e2310614. 38200684 10.1002/smll.202310614

[169] Zhang H, Deng S, Jin F, Qu J, Fan X, Chen P, Fan X. Organoids and organ-on-a-chip models for investigating the pathophysiology of the human reproductive system. Interdiscip Med. 2026;4(2): Article e70094.

[170] Ronaldson-Bouchard K, Teles D, Yeager K, Tavakol DN, Zhao Y, Chramiec A, Tagore S, Summers M, Stylianos S, Tamargo M, et al. A multi-organ chip with matured tissue niches linked by vascular flow. Nat Biomed Eng. 2022;6(4):351–371. 35478225 10.1038/s41551-022-00882-6 PMC9250010

[171] Huang Y, Wu X, Xu Y, Yang N, Xi P, Wang Y, Zhu Y, Chen X. Organoids/organs-on-chips towards biomimetic human artificial skin. Burns Trauma. 2025;13: Article tkaf029. 40740685 10.1093/burnst/tkaf029 PMC12309384

[172] Du C, Liu J, Liu S, Xiao P, Chen Z, Chen H, Huang W, Lei Y. Bone and joint-on-chip platforms: Construction strategies and applications. Small Methods. 2024;8(12): Article e2400436. 38763918 10.1002/smtd.202400436

[173] Kang L, Sheng J, Bao X, He G, Mao L, Wang Q, Lu J, Zhou M, Zhang Y, He L, et al. Establishment of the multi-organ circulatory and supervision system (mocs). Sci Bull. 2025;70(4):488–491. 10.1016/j.scib.2024.12.038 39743468

[174] Qiao L, Chang S, Zou L, Zhang F, Cui C, Huang N. Multiorgan-on-a-chip: An advanced platform for disease modeling, drug toxicity assessment, and therapeutic screening. Bio Des Manuf. 2025;8(6):1035–1062.

[175] Lin L, Wang X, Niu M, Wu Q, Wang H, Zu Y, Wang W. Biomimetic epithelium/endothelium on chips. Eng Regen. 2022;3(2):201–216.

[176] D’Alessandro A. Red blood cell omics and machine learning in transfusion medicine: Singularity is near. Transfus Med Hemother. 2023;50(3):174–183. 37434999 10.1159/000529744 PMC10331163

[177] Gou M-H, Yang H-S, Fang Y-G. Construction of a pregnancy prediction model in acupuncture treatment for diminished ovarian reserve based on machine learning. World J Acupunct Moxibustion. 2025;35(1):32–40.

[178] Yuan H, Yu K, Xie F, Liu M, Sun S. Automated machine learning with interpretation: A systematic review of methodologies and applications in healthcare. Med Adv. 2024;2(3):205–237.

[179] Yuan H. Overcoming computational resource limitations in deep learning for healthcare: Strategies targeting data, model, and computing. Med Adv. 2025;3(1):42–45.

[180] Li C, Xue J, Zhou D, Qi X, Chen T, Li Z. Integrating pharmacogenetics, multi-omics and machine learning in the novel therapeutic view of pulmonary hypertension. Interdiscip Med. 2025;3(1): Article e20240029.

[181] Tang J-W, Yuan Q, Wen X-R, Usman M, Tay ACY, Wang L. Label-free surface-enhanced Raman spectroscopy coupled with machine learning algorithms in pathogenic microbial identification: Current trends, challenges, and perspectives. Interdiscip Med. 2024;2(3): Article e20230060.

[182] Khan RU, Almakdi S, Alshehri M, Haq AU, Ullah A, Kumar R. An intelligent neural network model to detect red blood cells for various blood structure classification in microscopic medical images. Heliyon. 2024;10(4): Article e26149. 38384569 10.1016/j.heliyon.2024.e26149 PMC10879026

[183] Yang Y, He H, Wang J, Chen L, Xu Y, Ge C, Li S. Blood quality evaluation via on-chip classification of cell morphology using a deep learning algorithm. Lab Chip. 2023;23(8):2113–2121. 36946151 10.1039/d2lc01078j

[184] Tong Y, Zeng Y, Lu Y, Huang Y, Jin Z, Wang Z, Wang Y, Zang X, Chang L, Mu W, et al. Deep learning-enhanced microwell array biochip for rapid and precise quantification of cryptococcus subtypes. View. 2024;5(4): Article 20240032.

[185] Cui D, Zhou L, Hauser CAE, Li Z-A, Lei Y. The organ-on-a-chip–AI virtual cell loop: A path toward next-generation biomedicine. Sci Bull. 2026. 10.1016/j.scib.2026.07.080 42580899

[186] Piety NZ, Stutz J, Yilmaz N, Xia H, Yoshida T, Shevkoplyas SS. Microfluidic capillary networks are more sensitive than ektacytometry to the decline of red blood cell deformability induced by storage. Sci Rep. 2021;11(1):604. 33436749 10.1038/s41598-020-79710-3 PMC7804960

[187] Branch SA, Wang Y, Azibere S, Soule LD, Davis AR, McMahon T, Martin RS, Baker LA, Geiger MK, Spence DM. Microfluidic determination of cell-derived ATP and single cell pressure mapping confirms benefits of normoglycemic stored red blood cells. ACS Meas Sci Au. 2025;5(4):511–519. 40861914 10.1021/acsmeasuresciau.5c00032 PMC12371595

[188] Myers DR, Sakurai Y, Tran R, Ahn B, Hardy ET, Mannino R, Kita A, Tsai M, Lam WA. Endothelialized microfluidics for studying microvascular interactions in hematologic diseases. J Vis Exp. 2012;(64):3958. 22760254 10.3791/3958 PMC3471282

[189] Mathur T, Singh KA, Pandian NK, Tsai S-H, Hein TW, Gaharwar AK, Flanagan JM, Jain A. Organ-on-chips made of blood: Endothelial progenitor cells from blood reconstitute vascular thromboinflammation in vessel-chips. Lab Chip. 2019;19(15):2500–2511. 31246211 10.1039/c9lc00469f PMC6650325

